# Efficacy of Functional Foods, Beverages, and Supplements Claiming to Alleviate Air Travel Symptoms: Systematic Review and Meta-Analysis

**DOI:** 10.3390/nu13030961

**Published:** 2021-03-16

**Authors:** Virginia Chan, Leanne Wang, Margaret Allman-Farinelli

**Affiliations:** Nutrition and Dietetics Group, School of Life and Environmental Science, Charles Perkins Centre, The University of Sydney, Camperdown 2006, Australia; lwan4745@uni.sydney.edu.au (L.W.); margaret.allman-farinelli@sydney.edu.au (M.A.-F.)

**Keywords:** dietary supplements, functional food, functional beverage, jetlag syndrome, sleep

## Abstract

Airline passengers experience a range of symptoms when travelling on long flights. This review evaluated the efficacy of functional foods, beverages, and supplements claiming to address the effects of air travel for healthy adults. Products were identified in a scoping review of electronic databases, search engines, and grey literature (March to August 2019). A systematic review of the efficacy of product ingredients was conducted using five electronic databases from inception to February 2021. Articles were screened, data extracted, and assessed for risk of bias by two researchers independently. Meta-analysis was performed. Of the 3842 studies identified, 23 met selection criteria: melatonin (*n* = 10), Pycnogenol (*n* = 4), various macronutrients (*n* = 2), caffeine (*n* = 2), *Centella asiatica* (*n* = 1), elderberry (*n* = 1), *Echinacea* (*n* = 1), fluid (*n* = 1), and Pinokinase (*n* = 1). Meta-analysis (random effects model) indicated melatonin reduced self-reported jetlag following eastbound (*n* = 5) and westbound (*n* = 4) flights: standard mean difference −0.76 (95% CI = −1.06 to −0.45, I2 0%, *p* < 0.00001) and −0.66 (95% CI = −1.07 to −0.26, I2 45%, *p* = 0.001), respectively. Pycnogenol also reduced edema scores (*n* = 3), standard mean −4.09 (95% CI = −6.44 to −1.74), I2 98%, *p* = 0.0006). Overall, 12 of 183 ingredients contained in 199 products had evidence to support claims.

## 1. Introduction

The popularity of international travel has been growing, and over 4.3 billion passengers commuted by air in 2018 [1]. The increasing number of travelers exposed to long- and ultralong-haul flights can experience a range of physiological and psychological symptoms. Despite significant disruptions to international travel due to the COVID-19 pandemic, the rollout of vaccines suggests that long-haul international air travel may soon resume.

Jetlag, the desynchronization of normal circadian rhythm, occurs as a result of rapid travel through multiple time zones [2]. This is characterized by sleep disturbances, daytime fatigue, reduced cognitive and physical performance, and alterations in mood [2,3]. The severity of symptoms worsens following eastward travel over multiple time zones [2].

Air travel has also been associated with several other physical conditions: the sensation of abdominal distention and bloating due to expansion of gases within the gastrointestinal tract at typical aircraft cruising altitudes [4,5], dehydration from low cabin humidity, and the consumption of diuretic beverages commonly served onboard commercial flights such as alcohol. Additionally, the mildly hypoxic cabin conditions combined with dehydration and reduced physical activity result in an increased risk of deep vein thrombosis [5] and edema [6]. The close seating proximity of passengers over long-haul flights increases the possibility of transmission of infectious diseases [7]. Of some concern to cabin crews and frequent travelers is the prolonged exposure to radiation in long-haul flights, and the high altitude of flights may increase an individual’s radiation exposure [8].

Several pharmacological and non-pharmacological treatments exist to lessen the symptoms associated with flight. These include light therapy for jetlag [9] as well as the implementation of high-efficiency particulate air filters to improve cabin air quality [7,10].

The food and supplement industries have responded with a range of products that claim to address one or more of the unwanted symptoms associated with air travel. These products advertise a range of ingredients including melatonin and herbal extracts. However, they often do not provide peer reviewed scientific evidence conducted in in-flight settings or flight simulations to support claims made. Twenty percent of Australian airline passengers reported consumption of vitamins or dietary supplements, and 8% indicated use of melatonin to help cope with jetlag and travel fatigue [11].

An evidence-based approach is required to assist passengers, cabin crew, and airlines in their decision to purchase and consume such products. This study aimed to evaluate the efficacy of functional foods, beverages, and supplements that claim to address the effects of air travel for healthy adults.

## 2. Materials and Methods

This study was a two-stage process. The first phase was a scoping review of functional foods, beverages, and supplements that claimed to alleviate air travel related symptoms. The second stage was a systematic literature review of the evidence surrounding the health claims made by the products identified in the scoping review.

The methodology employed in this paper was developed using the Arksey & O’Malley’s Scoping Study Methodological Framework [12] and the Preferred Reporting Items for Systematic Reviews and Meta-analyses (PRISMA) framework [13], guiding stages 1 and 2, respectively. The details of this protocol were pre-published [14].

### 2.1. Scoping Review

The scoping review of available functional foods, beverages, and supplements was conducted from 6 March 2019 until 31 August 2019.

#### 2.1.1. Search Strategy

Functional foods, beverages, and supplements were identified using four databases (Medline, Embase, PsycINFO, and Web of Science) and two search engines (Google and Bing). Search terms were combinations, truncations, and synonyms of terms relating to food and beverages with aviation terms and symptoms associated with flight. Databases were searched from inception until April 2019. A sample search strategy for Medline (via Ovid) is provided in Supplementary Table S1. Due to the large number of records recalled on the Google and Bing search engines, only the first 15 pages from each of the 17 different searches per engine were reviewed according to the default display. The search terms are shown in Supplementary Table S2. Several grey literature publications (PAX International, APEX, and Onboard Hospitality) that circulate articles and advertisements of potentially relevant functional foods, beverages, and supplements were also hand-searched from earliest publicly available issue.

#### 2.1.2. Product Inclusion and Exclusion Criteria

Items identified were investigated further by an online search and were included in the product database if they were (i) stocked or marketed to airlines, airports, or commercial cabin crew; (ii) claimed to be used or developed by commercial cabin crew; (iii) had a scientific publication trialing the product within an air flight simulation setting; (iv) provided instructions for commercial inflight use.

Products were excluded from the database if they were discontinued at the time of the search or did not fulfill the inclusion criteria.

#### 2.1.3. Product Database Formation

Using publicly available material, the following information was recorded in a Microsoft Excel 2011 spreadsheet (Microsoft, Redmond, WA, USA): product information, health claims made, evidence provided by the manufacturer, ingredients, price (per serve in Australian dollars), recommended method of consumption, and nutritional information (per serve and per 100 g).

#### 2.1.4. Ingredient Synthesis of Products within the Database

All ingredients with advertised possible beneficial effects on symptoms relating to flight contained within products included in the database were extracted and categorized into the following categories: vitamins, minerals, macronutrients, and other. These ingredients as well as the consumption method of these products were examined further by systematic literature review.

### 2.2. Systematic Review

#### 2.2.1. Search Strategy

The search was conducted from journal inception to the 25 February 2021 in the following electronic databases: Cumulative Index to Nursing and Allied Health Literature (CINAHL), Cochrane Central Register of Controlled Trials (CENTRAL), Embase, Medline (including Pre-Medline), and PsycINFO. The reference list of included studies and scientific articles referenced by products in the database were hand-searched for additional records.

The search terms were both the common and scientific names of the ingredients contained in the products in the database combined with synonyms and truncations of aviation terms and the databases’ subject headings. The Scottish Intercollegiate Guidelines Network (SIGN) randomized controlled trial study filter [15] was adapted to capture nonrandomized controlled trials and applied to searches conducted in CINAHL, Embase, and Medline with assistance from an academic liaison librarian. The search strategy for Medline (via Ovid) can be found in Supplementary Table S4.

#### 2.2.2. Eligibility Criteria

The following randomized and non-randomized controlled trials were included in order to capture all articles with appropriate control groups according to the following criteria:

- Population: healthy adults aged over 18 years without pre-existing health conditions that would impact the primary outcome of the intervention.
- Intervention: the administration of a food, beverage, or nutritional supplement to participants at any time before, during, or after a commercial air flight or simulation.
- Comparator: an appropriate control or comparison group receiving no intervention, a placebo, or standard management and underwent the same air flight or simulation as the intervention group.
- Outcomes: any qualitative or quantitative measurement of physical or cognitive symptoms associated with air travel.

Studies were excluded if they were

- Conducted under military or space flight settings, as the conditions of speed and altitude are not comparable to commercial air travel.
- Examined a combination pharmacological (other than caffeine and melatonin) or non-pharmacological therapies, whereby the specific effect of the test food, beverage, or nutritional supplement could not be ascertained.
- Non-English texts.
- Full paper was not available.

#### 2.2.3. Study Selection

**Figure 1:**
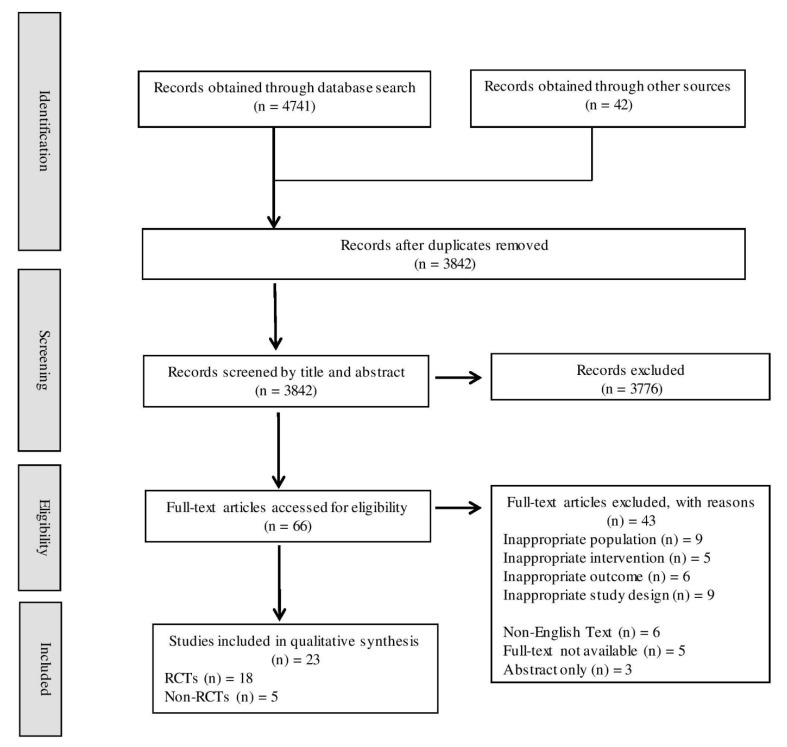
Flowchart of literature search and screening for selection of randomized and non-randomized controlled trials examining the effects ingredients found in functional foods, beverages, and supplements that claim to alleviate flight-related symptoms. Other sources included a hand search of reference lists of relevant systematic reviews as well as studies listed by products identified in scoping review.

Titles and abstracts were imported into the EndNote X9 reference management software (Clarivate Analytics, Philadelphia, PA, USA). Duplicates were removed, and titles and abstracts were reviewed by two independent assessors (VC and LW) against the eligibility criteria and assigned into two groups: (i) further review or (ii) excluded. The full text of potentially eligible papers was obtained and reviewed independently (VC and LW), and reasons for exclusion were recorded (Figure 1). Discrepancies in results were resolved through discussion and a third independent reviewer (MAF) involved when an agreement could not be reached.

#### 2.2.4. Data Extraction

The data extraction table designed was guided by the PRISMA statement [13] with some additional elements included. Two reviewers independently extracted the following data in duplicate: study details (author, year, country, funding, and affiliations), participants (characteristics, flight or simulation details, inclusion and exclusion criteria, attrition, and blinding), intervention and comparator details (intervention, sample size, length of intervention and follow-up, and retention rate), and outcomes (qualitative and quantitative measures of symptoms associated with flight and adverse effects).

#### 2.2.5. Data Synthesis and Analysis

The primary outcome of interest was the change in cognitive and physical symptoms associated with air flight. Studies were grouped according to their intervention (and flight direction if applicable). Where possible, for all study arms results were recorded as mean at baseline and post-intervention, with measures of error (SEM or SD) and associated *p*-values.

A meta-analysis was performed on the edema scores (3 trials) for Pycnogenol studies and visual analogue ratings of jetlag symptoms after melatonin administrations (5 trials eastbound and 4 trials westbound) for studies of similar design using Cochrane RevMan (version 5.4, The Cochrane Collaboration). A random effects model and standardized mean difference (Cohen d) was applied. Data were presented graphically as forest plots by intervention (and flight direction for melatonin). Studies were grouped according to the timing of administration of melatonin as either prior to flight or at bedtime on the day of or following flight. Asymmetry could not be assessed by funnel plot analysis as there were fewer than 10 studies included in the meta-analyses [16]. Missing data required for the meta-analysis were obtained from a previous publication [2] or imputed as per the Cochrane Systematic Review Handbook [16].

#### 2.2.6. Quality Assessment

Risk of bias was assessed independently by the two reviewers (VC and LW) using the appropriate Cochrane Collaboration Tool-Risk of Bias for Randomized Control Trials II [17] or Robins I for Other Non-Randomized Interventions [18].

Grading of Recommendations Assessment, Development and Evaluation system [19] was not performed as initially proposed in the published protocol [14]. This diversion from the protocol is explained in the Discussion section.

## 3. Results

### 3.1. Scoping Review

A total of 199 functional foods, beverages, and supplements were included in the database. Of which, 55.8% were unique (*n* = 111) and the remaining were the same product but of a different flavor (*n* = 47, 23.6%), portion size (*n* = 40, 20.1%), or part of a package (*n* = 1, 0.5%).

Of the unique products, beverages were the most common type (*n* = 38, 34.2%), followed by orally consumed capsules/tablets/concentrates (*n* = 32, 28.8%) and water-soluble tablets/powders/concentrates (*n* = 29, 26.1%). Confectionary (*n* = 5, 4.5%), bar/biscuit/cookies (*n* = 4, 3.6%), yoghurt (*n* = 1, 0.9%), nuts (*n* = 1, 0.9%), and fruit chips (*n* = 1, 0.9%) made up a smaller component of types of products identified.

Cumulatively, 302 health claims were made, where 93.7% of products made more than one statement. Improvements to feelings of fatigue (37.8%), immune response (36.9%), jetlag symptoms (32.4%), sleep (32.4%), hydration (27.9%), anxiety (26.1%), and cardiovascular health (21.6%) were the most common, as indicated in Table 1.

A total 183 ingredients were advertised to deliver the improvements to health and wellbeing of these products. As shown in Table 2, the majority of products (66.7%) promoted at least one herbal compound or supplement as an ingredient purporting to have beneficial effects. Pycnogenol was reported in 6.3% of all products, 2.7% for caffeine, and 12.6% for melatonin. However, 24.3% of products identified did not provide a full ingredient list.

The majority of evidence to support claims made by products was primarily in the form of generalized statements with no peer reviewed publications referenced (79.3%) and lay testimonies from consumers (54.1%). A small proportion of products (*n* = 15) provided some form of scientific evidence, most of these were not conducted within flight settings (*n* = 6) or did not have results published (*n* = 2) at the time that the scoping review was completed. Of the remaining products, results were published in the form of a letter (*n* = 1), subsection of a review paper (*n* = 1), or conference abstracts (*n* = 3). Only 2 out of the 199 products referenced peer reviewed publications of studies that were conducted within appropriate air flight settings.

The price of each serve ranged between $0.18 to $14.95 Australian dollars, with an average of $3.20 (SD ± 2.30, *n* = 69) for products with cost listed. Products were mostly from the United States of America (*n* = 29), United Kingdom (*n* = 16), Netherlands (*n* = 12), New Zealand (*n* = 11), Australia (*n* = 7), and Turkey (*n* = 7). The remaining products originated from Canada, Portugal, Austria, Denmark, Sweden, Germany, Ireland, Switzerland, Israel, Japan, and Thailand.

### 3.2. Systematic Review

#### 3.2.1. Study Selection

As shown in Figure 1, a total of 4741 articles were obtained by database searching. A further 42 studies were identified through hand searching reference lists and articles referenced by products included within the scoping review database. After removal of duplicates, 3842 records were screened by title and abstract, of which 3776 were excluded. The remaining 66 studies underwent full-text review. Twenty-three studies met the eligibility criteria and were included in this review. The remaining 43 studies were excluded with reasons provided (Figure 1 and Supplementary Table S3).

#### 3.2.2. Characteristics of All Included Studies

The 23 studies included are summarized in Table 3 and Table 4 and examined 12 different ingredients: caffeine (*n* = 2), *Centella asiatica* (*n* = 1), *Echinacea* (*n* = 1), elderberry (*n* = 1), fluid (*n* = 1), fiber (*n* = 1), macronutrients (*n* = 1), melatonin (westbound *n* = 1, eastbound *n* = 6, and both directions *n* = 3), Pinokinase (*n* = 1), and Pycnogenol (*n* = 4).

**Table 3:** Summary of included studies and demographics of participating populations conducted within (**a**) flight settings and (**b**) simulated flight settings.

(a)						
Author, Year, Study Design ^1^	Agent ^1^	Flight Conditions ^1^	Trial Arms (*n*) ^1^	Participant Characteristics ^1^	Intervention Description ^1^	Duration ^1^
Cesarone et al., 2001, RCT [20]	*Centella asiatica*	Commercial air flight (economy class) Length: 3–14 h Direction: NR Country: NR	*n* = 66 I = 33 C = 33 Power: NR	Age: range 30–50 years Gender: 50% male No previous deep vein thrombosis or flight within the previous 7 days	Two days prior to flight until one day post flight, participants consumed: I = 60 mg Centellase tablet three times per day C = no drug or other treatment, no further information provided. Unclear if participants were blinded	Follow up period: <4 h post flight % followed up: 91% Excluded: dropped out (*n*) = 6, group NR Compliance: 97%
Tiralongo et al., 2016, RCT [21]	Elderberry	Commercial air flight (economy class) Length: 7+ h Direction: NR Country: Australia	*n* = 325 I = 163 C = 162 Power: *n* = 140 per intervention arm (α ≤ 0.05, β ≥ 0.80)	Age: mean (SD) 51 (16) years Gender: 44% male Good general health with no known plant allergy or existing respiratory disease; 54% received flu vaccination.	10–2 days prior to flight consumed 2 capsules/day and 1 day prior to flight until 4–5 days after arrival at destination consumed 3 capsules/day that contained: I = 300 mg/capsule elderberry extract C = placebo, no further information Participants were blinded	Follow up period: 4–5 days post flight % followed up: 87% Excluded: Discontinued (*n*): I = 10, C = 9 No intervention (*n*): I = 5, C = 8 Lost to follow up (*n*): I = 4, C = 6 Compliance: 60%
Tiralongo et al., 2012, RCT [22]	*Echinacea*	Commercial air flight (economy class) Length: 15–25 h Direction: NR Country: Australia	*n* = 170 I = 85 C = 85 Power: *n* = 180 (α = 0.05, β = 0.80)	Age: mean (SD) 43 (14) Gender: 33% male Good general health with no known plant allergy, existing respiratory disease	14–3 days before flight: 1 tablet twice/day. 2 days prior to flight until 7 days post arrival at destination: 2 tablets twice/day. 8 days post arrival until 3 days prior to return flight: 1 tablet twice/day. 2 days prior to return flight until 7 days post return: 2 tablets twice/day. 8–14 days post return 1 tablet twice/day. I = Echinacea (112.5 mg *Echinacea purpurea* and 150 mg *Echinacea angustifolia*) C = placebo Note: Sick dose 3 tables twice a day Participants were blinded	Follow up period: 4 weeks post return flight % followed up: 82% Excluded: Excluded due to various reasons (*n*): I = 3, C = 2 No intervention (*n*): I = 3, C = 2 Lost to follow up (*n*): I = 17, C = 10 Compliance: I = 93% and C = 95%
Arendt et al., 1988, cross over-RCT [23]	Melatonin	Commercial air flight Length: NR Direction: Both Country: UK, Australia or New Zealand	*n* = 61 I = 57 C = 56 Power: NR	Age: NR Gender: 72% male	2 days prior to flight until day prior to arrival 1 tablet at 2 am destination time. Day of arrival until 4 days post arrival 1 tablet at local bedtime. Stay: >14 days Return protocol: repeated with the other intervention arm I = 5.0 mg melatonin C = placebo Participants were blinded	Follow up: 7 days post flight % followed up: 85% Excluded: (*n*): I = 5, C = 4, only completed single flight direction excluded from within subject comparison Compliance: NR
Nickelsen et al., 1991, non-RCT [24]	Melatonin	Commercial air flight Length: 6–11 h Direction: Both Country: West Germany or North America	*n* = 36 I = 18 C = 18 Power: NR	Age: mean (SD) 26 (3) years Gender: 72% male	Following westbound flight: 1 capsule for 7 days at bedtime. Participants stayed at for >14 days. Following eastbound flight: 1 capsule for 5 days at bedtime I = 5.0 mg melatonin C = placebo Participants were blinded	Follow up: 7 and 5-days post west- or eastbound flights respectively: % followed up: NR Excluded: NR Compliance: NR
Petrie et al., 1989, Cross Over RCT [25]	Melatonin	Commercial air flight Length: 26 h Direction: Both Country: New Zealand or UK	*n* = 20 I = 20 C = 20 Power: NR	Age: range 26–68 Gender: 60% male	3 days prior to flight and day of flight: capsule at 10:00–12:00 local time. 1–3 days post arrival: capsule at 22:00–24:00 destination time. Stay: 3 weeks. Return protocol: repeated with the other arm. I = 5.0 mg melatonin C = placebo Participants were blinded	Follow up: 10 days post flight % followed up: 100% Excluded (*n*) = 0 Compliance: NR
Petrie et al., 1993, RCT [26]	Melatonin	Commercial air flight Length: NR Direction: West Country: UK	*n* = 52 I_1_ = 14 * I_2_ = 15 * C = 15 * * included in day 6 analysis Power: NR	Age: mean (SD) 35 (8) years Gender: 50% male Air New Zealand Cabin Crew rostered on same 9-day duty. Return trip from New Zealand to UK. Study completed on westbound return journey	2 days prior to flight: 2–3 a.m. NZST Day of flight: 12 p.m. NZST 1–5 days post arrival: 10–12 pm NZST I_1_ = 5.0 mg melatonin capsule I_2_ = 0.5 mg melatonin (+placebo on 2 days prior to flight) capsules C = placebo capsule Participants were blinded	Follow up: 6 days post flight % followed up: 85% Excluded: (*n*) = 8, final questionnaire not completed group NR Compliance: NR
Arendt et al., 1987, RCT [27]	Melatonin	Commercial air flight Length: NR, 8 time zones crossed Direction: East Country: America	*n* = 17 I = 8 C = 9 Power: NR	Age: mean (SEM) 49 (2) years Gender: 41% male Good general health Return trip from London to Los Angeles with 2 weeks stay. Study completed on eastbound return journey.	2 days prior to flight and day of flight: 18.00 h local time 1–4 days post arrival: bedtime I = 5.0 mg melatonin capsule C = placebo capsule Participants were blinded	Follow up: 22 days post flight % followed up: 100% Excluded: no jetlag symptoms (*n*): C = 2 Compliance: NR
Claustrat et al., 1992, non-RCT [28]	Melatonin	Commercial air flight Length: NR Direction: East Country: North America	*n* = 37 I = 20 C = 20 * *n* = 3 cross over Power: NR	Age: NR Gender: 49% male Good general health Return trip from Lyon to North America with minimum 1 week stay. Study completed on eastbound return journey.	Day of flight: 22-n hours (where n is time lag between departure and destination) 1–3 days post flight: 10–11 pm local time I = 8.0 mg melatonin capsule C = placebo capsule Participants were blinded	Follow up: 7 days post flight % followed up: 72% Excluded: dropped out, (*n*): I = 5, C = 5, reasons NR but not due to side effects Compliance: NR
Edwards et al., 2000, RCT matched pairs [29]	Melatonin	Commercial air flight Length: 24 h Direction: East Country: UK	*n* = 34 I = 17 C = 17 Power: NR	Age: mean (SD) I = 41 (13) years, C = 41 (12) years Gender: 90% male (participants included)	Day of flight: 18:00–19:00 local time 1–4 days post arrival: 22:00–23:00 local time I = 5.0 mg melatonin capsule C = placebo capsule Participants were blinded	Follow up: 6 days post flight % followed up: 82% Excluded: Illness (*n*): I = 3 Incomplete dataset (*n*): C = 3 Compliance: NR
Spitzer et al., 1999, RCT [30]	Melatonin	Commercial air flight Length: 6 h Direction: East Country: America	*n* = 257 * I_1_ = 64 * I_2_ = 70 * I_3_ = 63 * C = 60 * * completersPower: NR	Age: mean (SD) 44 (7) years * Gender: 79% male * Attendees of a pharmaceutical-company-sponsored educational program. * completers Return trip from Norway to New York with 5 days stay. Study conducted on return trip	Day of flight until 5 days post arrival, participants consumed capsules: I_1_ = 5.0 mg melatonin at bedtime I_2_ = 0.5 mg melatonin at bedtime I_3_ = 0.5 mg melatonin 11 h after wake C = placebo Cointerventions: sleep mask on airplane, alcohol avoidance, no sleep medication Participants were blinded	Follow up: 6 days post flight % followed up: 76% Excluded: Noncompleters (*n*) = 82, reason and group NR Compliance: NR
Suhner et al., 2001, RCT [31]	Melatonin	Commercial air flight Length: mean (SD) 12 (4) hours, 6–9 time zones crossed Direction: East Country: America	*n* = 160 I_1_ = 40 I_2_ = 40 I_3_ = 40 C = 40 Power: NR	Age: mean (SD) 41 (NR) years * Gender: 51% male * Return trip from Switzerland to America with minimum 1 week stay. Study completed on eastbound return journey. * completers	Day of flight: 1700–2100 departure time 1–4 days post arrival at local bedtime I_1_ = 5.0 mg melatonin + placebo capsules I_2_ = 10.0 mg zolpidem + placebo capsules I_3_ = 5.0 mg melatonin + 10.0 mg zolpidem capsules C = placebo + placebo capsules Participants were blinded	Follow up: 4 days post flight (with 4-day baseline measurement 2 weeks post flight) % followed up: 86% Excluded: Noncompliant (*n*) = 9, group NRAdverse effects (*n*) = 14, group NR Compliance: NR
Suhner et al., 1998, RCT [32]	Melatonin	Commercial air flight Length: NR, 6–8 time zones crossed Direction: East Country: Switzerland or America	*n* = 320 I_1_ = 80 I_2_ = 80 I_3_ = 80 C = 80 Power: NR	Age: mean (SD) 36 (NR) years Gender: 54% male Good general health	1–4 days post arrival at local bedtime I_1_ = 0.5 mg fast release melatonin I_2_ = 5.0 mg fast release melatonin I_3_ = 2.0 mg controlled release melatonin C = placebo Participants were blinded	Follow up: 4 days post flight % followed up: 73% Excluded: Noncompliant (*n*) = 75, group NR Withdrew (medical reasons) (*n*) = 2, group NR Travel illness (*n*) = 9, group NR Compliance: 77%
Cesarone et al., 2003, RCT [33]	Pinokinase	Commercial air flight Length: 7–8 h Direction: Both Country: UK or America	*n* = 224 I = 110 C = 114 Power: NR	Age: mean (SD) I = 48 (12) years, C = 50 (13) years. * Gender: 51% male * High risk of deep vein thrombosis but no recent thrombosis (<6 months) * completers	2 capsules with 250 mL water 2 h prior to flight, repeated 6 h later. I = 150 mg Pinokinase (per capsule, 300 mg total dosage) C = Placebo * SM: exercise and regular water drinking Unclear if participants were blinded	Follow up: acute % followed up: 83% Excluded: Drop out (*n*) = 18 poor compliance or flight connections, group NR Unclear reason (*n*) = 20, group NR Compliance: NR
Belcaro et al., 2018, non-RCT [34]	Pycnogenol	Commercial air flight (economy class) Length: 8+ h Direction: NR Country: NR	*n* = 295 I = 90 C_1_ = 99 C_2_ = 106 Power: NR	Age: NR Gender: 52% male Participants of varying risk of edema and DVT but no recent thrombosis (<6 months)	3 days prior to flight until 3 days post flight: I = 50 mg Pycnogenol capsule three times per day (150 mg total dosage) C_1_ = SM C_2_ = SM + compression stockings * SM: exercise and regular water drinking Participants were not blinded	Follow up: acute % followed up: 100% Excluded (*n*) = 0 Compliance: NR
Belcaro et al., 2008, non-RCT [35]	Pycnogenol	Study 1: Commercial air flight (economy/business) Length: 10–14 h Direction: West Country: NR	*n* = 68 I = 38 C = 30 Power: NR	Age: mean (SD) I = 48 (12) years, C = 45 (7) years * Gender: 57% male * Subgroup: mild hypertension treated with anti-hypertensive medication * completers	2 days prior to flight until 4 days post arrival: I = 50 mg Pycnogenol capsules 3 times per day (150 mg total dosage) C = NR Unclear if participants were blinded	Follow up: 48 h % followed up: 88% Excluded: Non-medical issues or loss of contact (*n*) = 8, group NR Compliance: NR
		Study 2: Commercial air flight (economy/business) Length: 7–9 h Direction: NR Country: NR	*n* = 65 I = 34 C = 31 Power: NR	Age: average 54 (6) years Gender: 52% male Subgroup: mild hypertension treated with anti-hypertensive medication		Follow up: 28 h % followed up: 92% Excluded: Non-medical issues or loss of contact (*n*) = 5, group NR Compliance: NR
Belcaro et al., 2004, RCT [36]	Pycnogenol	Commercial air flight Length: 7–12 h Direction: NR Country: NR	*n* = 244 I = 110 C = 114 Power: NR	Age: NR Gender: NR Moderate-high risk of DVT but no recent thrombosis (<6 months)	2 capsules with 250 mL water 2–3 h prior to flight, repeated 6 h later. 1 capsule the following day. I = 100 mg Pycnogenol (per capsule, 200 mg total dosage) C = Placebo Unclear if participants were blinded	Follow up: <2 h % followed up: 81% Excluded: lost at end of flight (*n*) = 13, group NR non-medical reasons (*n*) = 33, group NR Compliance: NR
Cesarone et al., 2005, non-RCT [37]	Pycnogenol	Commercial air flight Length: 7–12 h Direction: NR Country: NR	*n* = 211 I = 106 C = 105 Power: NR	Age: average (SD) 45 (8) years Gender: NR No recent thrombosis (<6 months)	2 capsules with 250 mL water 2–3 h prior to flight, repeated 6 h later. 1 capsule the following day. I = 100 mg Pycnogenol (per capsule) C = Placebo Unclear if participants were blinded	Follow up: acute % followed up: 80% Excluded: reasons NR (*n*): I = 25, C = 17 Compliance: NR
**(b)**						
**Author, Year, Study Design ^1^**	**Agent ^1^**	**Flight Conditions ^1^**	**Trial Arms (*n*) ^1^**	**Participant Characteristics ^1^**	**Intervention Description ^1^**	**Author, Year, Study Design ^1^**
Caska et al., 2007, RCT [38]	Caffeine	Computer simulation Length: 10 min Direction: NR Country: Australia	*n* = 30 I_1_ = 10 I_2_ = 10 C = 10 Power: NR	Age: mean (SD) 23 (4) years Gender: NR Held current Class 1 Aviation Medical Certificate and abstained from caffeine for 6 h.	Consumed a lemon-based solution after baseline measurement that contained: I_1_ = 1.0 mg/kg of body weight caffeine I_2_ = 3.0 mg/kg of body weight caffeine C = 0.0 mg/kg caffeine Participants were blinded	Caska et al., 2007, RCT [38]
Dagan et al., 2006, Cross over RCT [39]	Caffeine	Computer simulation Length: 15 min Direction: NR Country: NR	*n* = 24 I_1_ = 24 I_2_ = 24 C = 24 Power: NR	Age: range 25–31 years Gender: 100% male No prior experience operating flight simulator	Consumed 1 pill at 23.00 h that contained: I_1_ = 200 mg modafinil I_2_ = 200 mg caffeine C = 200 mg starch Washout period: 2 weeks Participants were blinded	Dagan et al., 2006, Cross over RCT [39]
Lindseth et al., 2013, Cross Over RCT [40]	Fluid	Computer simulation Length: 20 min Direction: NR Country: America	*n* = 40 I = 40 C = 40 Power: *n* = 35 (α = 0.05, β = 0.80)	Age: mean (SD) 20 (2) years Gender: predominately male, no further information Third term in collegiate aviation program	2-week fluid diet, no alcoholic beverages and caffeine limited to <90 mg/day. I = high fluid (>80 ounces) C = low fluid (<40 ounces) Washout period: 2 weeks Unclear if participants were blinded	Lindseth et al., 2013, Cross Over RCT [40]
Hinninghofen, et al., 2006, Cross over RCT [4]	Fiber	Altitude Simulation Length: 8 h Direction: NR Location: NR	*n* = 16 I = NR C = NR Power: NR	Age: mean (SD) 26 (6) years Gender: 100% male Good general health and no history of gastrointestinal dysfunction	Overnight fasted subjects consumed test meal within 10 min: I = high fiber (20 g) C = low fiber (2 g) Washout period: separate days Participants were blinded for altitude but not for fiber content of test meal	Hinninghofen, et al., 2006, Cross over RCT [4]
Lindseth et al., 2011, Cross over RCT [41]	Macro-nutrients	Computer simulation Length: 20 min Direction: NR Country: America	*n* = 45 I_1_ = 45 I_2_ = 45 I_3_ = 45 C = 45 Power: *n* = 35 (α = 0.05, β = 0.80)	Age: mean (SD) 21 (2) years Gender: NR Participants held current federal aviation administration medical certificates and was in their third semester of commercial plot aviation course	4-day diet consisting of: I_1_ = high carbohydrate (56% carbohydrate, 22% fat, 22% protein) I_2_ = high protein (56% protein, 22% carbohydrate, 22% fat) I_3_ = high fat (56% fat, 22% carbohydrate, 22% protein) C = control diet (50% carbohydrate, 35% fat, and 15% protein) Washout period: 2 weeks Participants were blinded	Lindseth et al., 2011, Cross over RCT [41]

^1^ Abbreviations used: randomized controlled trials (RCT), non-randomized controlled trial (non-RCT), not reported (NR), standard deviation (SD), standard management (SM), intervention group (I), control/comparator group (C).

The characteristics of the interventions categorized by the two different test conditions are reported in Table 3 for studies conducted within flight conditions and Table 3 for studies conducted in simulated flight conditions.

#### 3.2.3. Characteristics of Studies Conducted within Flight Settings

The 18 studies conducted within flight settings are summarized in Table 3. These papers studied the effects of *Centella asiatica* [20], elderberry [21], *Echinacea* [22], melatonin [23,24,25,26,27,28,29,30,31,32], Pinokinase [33], and Pycnogenol [34,35,36,37]. Length of flight was reported in 12 papers and ranged from 3 to 26 h. Direction of flight was reported in 11 papers; 6 of which were eastbound, 1 westbound, and 4 were conducted in both directions. Two papers reported performing a power calculation [21,22] for sample size.

Most studies were RCTs (*n* = 13). Ten papers examined the effects of melatonin of which eight were RCTs [23,25,26,27,29,30,31,32] and two were non-RCTs [24,28]. Four articles examined the effects of Pycnogenol of which one was an RCT [36] and three were non-RCTs [34,35,37].

Participant characteristics and intervention procedures varied across studies. All supplements were administered in the form of a tablet, pill, or capsule. The results were collected <2 h following their flight (*n* = 4) [33,34,36,37] or participants were followed up over a period of 28 h to four weeks. The percentage of participants followed up was reported by 17 studies and ranged between 72% and 100%. The compliance of participants to allocated intervention was only reported in four studies [20,21,22,32] and ranged between 60% and 97%.

#### 3.2.4. Characteristics of Studies Conducted in Simulated Flight Settings

The five studies conducted in a simulated flight setting are outlined in Table 3. These interventions studied the effects of caffeine [28,39], fluid [40], fiber [4], and macronutrients [41]. Four studies used computer simulations [38,39,40,41], and one used a simulated flight altitude [4]. The length of flight simulations was reported in all studies and ranged from 10 min to 8 h.

All studies were RCTs. The interventions were administered in food form [4,41], liquid form [38,40], or as a tablet/pill [39]. Two studies reported performing a power calculation to determine sample size [40,41]. Participant characteristics and intervention procedures varied across studies. The results were collected for all participants immediately following the flight simulation. Full compliance with procedures (100%) was reported in three interventions [4,38,39].

The key outcomes are reported according to the test conditions: Table 4 details the characteristics of studies conducted within flight conditions and Table 4 for studies conducted in simulated flight conditions.

#### 3.2.5. Key Outcomes of Studies Conducted within Flight Settings

The key outcomes of studies conducted within flight settings are outlined in Table 4. Supplementation with *Centella asiatica* appeared to reduce the rate of ankle swelling and edema [20]. Pinokinase seemed to reduce the edema score and incidence of deep vein thrombosis in high-risk subjects [33]. One of the four studies examining the effects of Pycnogenol reported a reduction in the incidence of deep vein thrombosis and superficial venous thrombosis [36]. Elderberry supplementation did not seem to reduce the incidence of cold diagnosis; however, it appeared to reduce symptom score [21]. Administration of *Echinacea* reported a reduction in participants reporting respiratory illness [22].

Adverse effects were reported for studies examining the effects of elderberry [21], *Echinacea* [22], and melatonin [23,26,28,29,30,31,32] and included cold-like symptoms, tingling of the tongue and mouth, and headaches.

#### 3.2.6. Key Outcomes of Studies Conducted within Simulated Flight Settings

They key outcomes of studies conducted in simulated flight settings are summarized in Table 4. One of the studies studying caffeine found possible beneficial effects during sustained wake times [39]. The other study found no significant effects of caffeine dosage on flight performance [38]. Participant scores appeared to be negatively impacted by dehydration but not significantly influenced by high- or low-fluid diets [40]. Participants on a high-fat or high-carbohydrate diet seemed to achieve better flight scores than those consuming a high-protein diet [41].

Higher-fiber diets appeared to be associated with slower gastric emptying and higher reported gastrointestinal symptoms in flight altitude simulations when compared to participants on low-fiber diets [4].

Adverse effects were only reported for the study examining fiber on gastrointestinal symptoms [4], and no other adverse effects were reported by the other studies conducted in simulated flight settings.

#### 3.2.7. Impact of Melatonin on Self-Reported Jetlag Following Westbound Travel

Four papers (five interventions) [23,24,25,26] studied the effects of melatonin on jetlag symptoms in westbound travel (*n* = 3 both directions and remaining intervention in westward direction only) using visual analogue scales (see Table 4). The dosage of melatonin ranged between 0.5 mg and 5.0 mg and was administered prior to departure (*n* = 3) or at bedtime upon arrival (*n* = 1).

**Figure 2:**
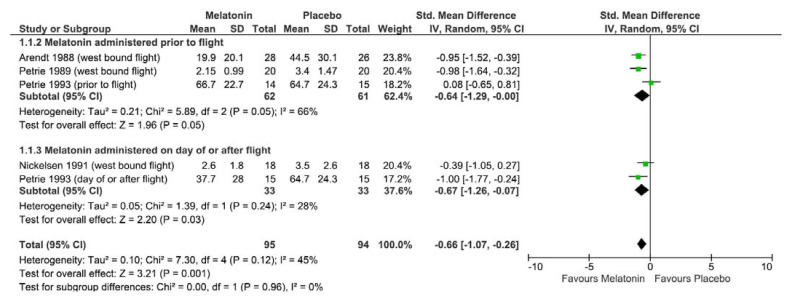
Forest plot of Cohen d effect size (standard mean difference) of studies examining the effect of melatonin on participant self-reported jetlag symptoms following westbound commercial air flights using a random effects model. Studies grouped by melatonin administration time of either prior to departure or at bedtime on the day of flight or after arrival. Diamond represents overall effect size, squares indicate percentage weighting of each study to overall effect size and 95% confidence intervals shown using horizontal lines.

The meta-analysis was performed on all four studies (five interventions). A random effects model was used to combine studies examining the effect of melatonin on self-reported jetlag following westbound flight. Figure 2 presents the forest plot, and the overall effect size between intervention and placebo group was −0.66 (95% CI = −1.07 to −0.26, I2 45%, *p* = 0.001). Subgroup analysis between administration prior to flight (effect size: −0.64, 95% CI = −1.29 to 0.00, I2 66%, *p* = 0.05) and on day of or after flight (effect size: −0.67, 95% CI = −1.26 to −0.07, I2 28%, *p* = 0.03) showed similar trends.

#### 3.2.8. Impact of Melatonin on Self-Reported Jetlag Following Eastbound Travel

Nine interventions examined the effect of melatonin on jetlag symptoms following eastbound travel (*n* = 3 both directions [23,24,25] and *n* = 6 in eastward direction only [27,28,29,30,31,32]). Seven interventions assessed jetlag with a visual analogue scale [23,24,25,26,27,28,29,31]; one used the Columbia jetlag scale [30]; the other asked about jetlag symptoms on a three-point scale [32]. One study applied the Liverpool jetlag questionnaire in addition to the visual analogue scale [29]. Dosage of melatonin varied between studies ranging from 0.5 mg to 8.0 mg. Administration schedules of melatonin differed between study protocols with studies providing melatonin to participants prior to departure (*n* = 3) or only at bedtime (*n* = 6) on the day of flight or after arrival.

**Figure 3:**
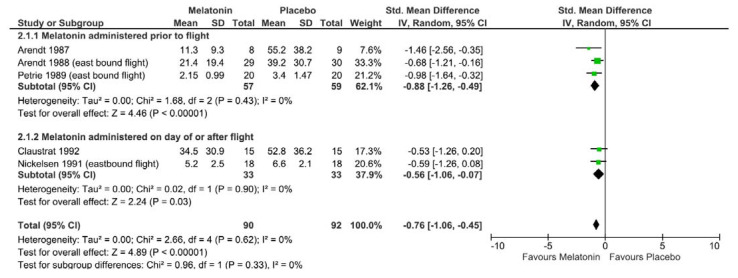
Forest plot of Cohen d effect size (standard mean difference) of studies examining the effect of melatonin on participant self-reported jetlag symptoms following eastbound commercial air flights using a random effects model. Studies grouped by melatonin administration time of either prior to departure or at bedtime on the day of flight or after arrival. Diamond represents overall effect size, squares indicate percentage weighting of each study to overall effect size and 95% confidence intervals shown using horizontal lines.

A meta-analysis was completed on five comparable studies, but four had to be excluded because the mean and variance measures were not reported or able to be calculated [29,30,31,32]. Authors were not contacted for additional information, as studies were published over 19 years prior to this review. A random effects model was used to combine studies examining the effect of melatonin on self-reported jetlag following eastbound travel. Figure 3 presents the forest plot, and the overall effect size between intervention and placebo group was −0.76 (95% CI = −1.06 to −0.45, I^2^ = 0%, *p* < 0.00001). Subgroup analysis between melatonin administration prior to flight (effect size: −0.88, 95% CI = −1.26 to −0.49, I^2^ 0%, *p* < 0.00001) and on the day of or after flight (effect size: −0.56, 95% CI = −1.06 to −0.07, I^2^ 0%, *p* = 0.03) showed similar trends.

#### 3.2.9. Impact of Pycnogenol on Edema

Three studies investigated the impacts of Pycnogenol on edema [34,35,37]. Dosages ranged from 100 mg to 200 mg and were administered within 2–3 days (*n* = 2) [34,35] or 3 h of departure (*n* = 1) [37]. Standard management of regular water consumption and exercise was employed in one study in all intervention groups [34].

**Figure 4:**
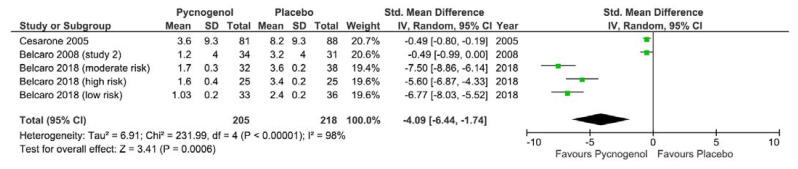
Forest plot of Cohen d effect size (standard mean difference) of studies examining the effect of Pycnogenol on participant edema following commercial air flights using a random effects model. Diamond represents overall effect size, squares indicate percentage weighting of each study to overall effect size and 95% confidence intervals shown using horizontal lines.

The meta-analysis was performed on all three studies. One study [34] provided sub-group results according to participant risk of developing edema and deep vein thrombosis i.e., low, moderate, or high risk. A random effects model was used to combine studies examining the effect of Pycnogenol on edema. Figure 4 presents the forest plot, and the effect size between intervention and placebo group was −4.09 (95% CI = −6.44 to −1.74, I2 98%, *p* = 0.0006).

#### 3.2.10. Risk of Bias of Included Studies

**Figure 5:**
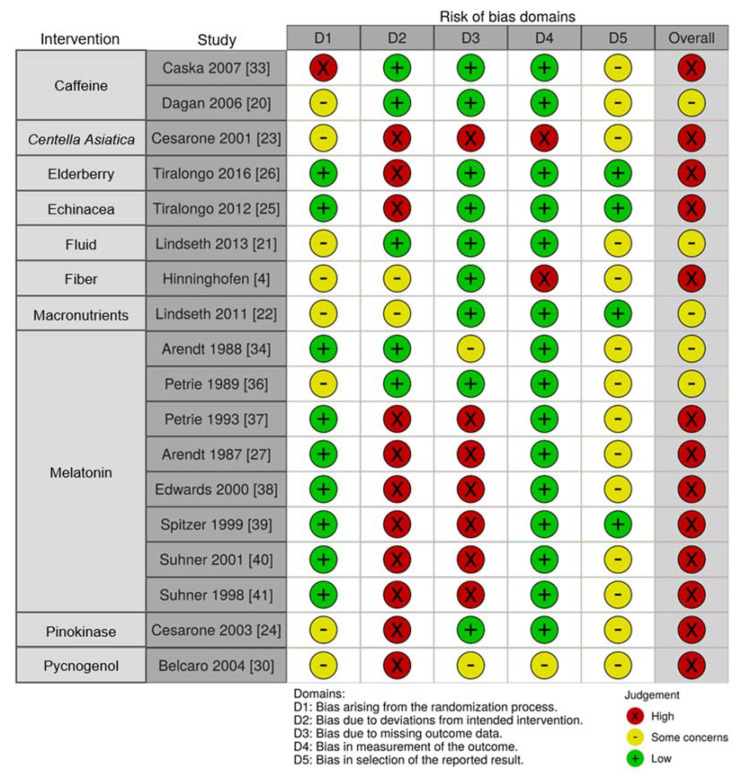
Risk of bias of randomized controlled trials assessed using Cochrane’s Rob-2 Tool presented according to the intervention agent as indicated [4,20,21,22,23,24,25,26,27,30,33,34,36,37,38,39,40,41].

The majority of RCTs were rated as high risk of bias (*n* = 13) as many did not report performing intention to treat analysis (domain 2) nor manage missing data appropriately (domain 3), as shown in Figure 5. Reporting bias could not be accurately discerned as only two of the RCTs provided details of their trial registration (domain 5).

**Figure 6:**
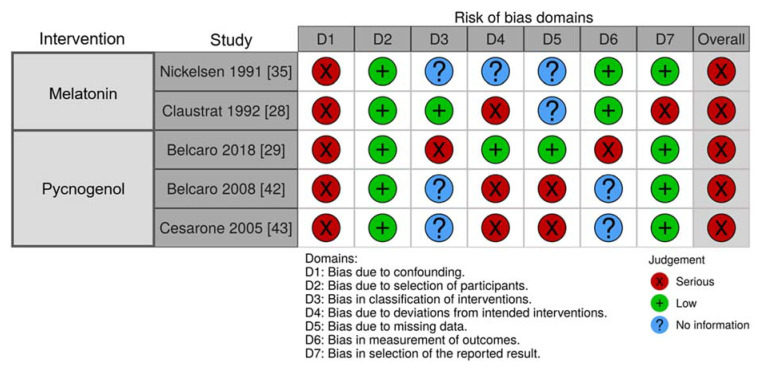
Risk of bias of randomized controlled trials assessed using the Cochrane’s Robins I Tool presented according to intervention agent as indicated [24,28,34,35,37].

Similarly, most non-RCTs were rated as serious risk of bias, as many did not adjust for confounding variables adequately (domain 1) nor report methodology to handle missing data (domain 5), indicated in Figure 6. Additionally, three studies had missing participants that were not accounted for in their analysis.

## 4. Discussion

A range of functional foods, beverages, and supplements was identified with 111 unique products included in the database as part of the scoping review, 93.7% of which made one or more health claims. Limited evidence was found to support claims made for such products. Only 12 out of 183 ingredients had scientific evidence trialing their use in flight settings or simulations across the 23 studies identified in the systematic review.

Melatonin had the greatest number of studies. Melatonin appears to have beneficial effects on self-reported jetlag following both east- and westbound flights. However, timing of melatonin ingestion may play a role because administration prior to flight appeared more effective than when administered on the day of or post flight. Travelers may have experienced more beneficial effects of the preflight melatonin following eastbound travel as it is often reported to be more severe than in the westerly direction [10]. This review employed a random effects model using standardized mean difference as study protocols, and outcome measurement was different between studies. Despite differences in methodology, the meta-analyses findings of this review for melatonin parallel those of a similar Cochrane review [2], which utilized a fixed effect model with mean difference. The meta-analysis of the effects of melatonin in this review also included the results of two additional papers in the westbound [25,26] and one in the eastbound [25] direction.

All studies included in the melatonin meta-analyses used a visual analogue scale to assess jetlag as these research studies were conducted prior to the development of the other measurement methods i.e., Liverpool scale (2000 [42]) or the Columbia scale (1999 [30]). A single subjective rating of jetlag on a visual analogue scale has limitations but remains useful for capturing the passengers’ experience of jetlag [43].

Most studies examining melatonin were classified as having a high risk of bias. Those employing an RCT design often did not report performing intention to treat analysis nor manage missing data appropriately. Of the two studies that were classified as non-RCT, they did not control for confounding variables.

Pycnogenol supplementation had favorable effects on edema. However, the majority of the studies examining this compound appear to have originated from the same group. No studies examining Pycnogenol adequately reported if their participants were blinded to intervention arm nor control for confounding variables (for non-randomized controlled interventions). They also introduced selection bias as they did not use intention to treat analysis for the RCTs. Despite the meta-analysis showing beneficial effects, it should be noted that due to the high level of heterogeneity (for Pycnogenol), a limited number of studies, and high risk of bias, the results should be interpreted with caution.

The remaining 14 studies examined the effects of caffeine, *Centella asiatica*, elderberry, *Echinacea*, Pinokinase, and diets containing various levels of fluid, macronutrients, or fiber. All interventions only had one study examining its effects, with the exception of caffeine that had two studies. None of these ingredients were tested under both flight and simulation conditions.

The majority of studies were rated as high risk of bias, and consequently the results of the included papers should be interpreted with caution. There is an insufficient evidence base to make a definitive judgement for their usage within air flight settings.

In Australia, the efficacy of low-risk complementary medicines is not assessed prior to sale, and products that are typically considered food can in some instances make health claims and not be classified as a therapeutic good [44,45]. This reflects why the majority of the products identified in this study did not provide high-quality scientific evidence to justify the health claims made.

Most interventions were well tolerated with no adverse effects reported by participants. Supplementation with elderberry resulted in some reports of cold and flu symptoms. Those administered with *Echinacea* reported vomiting, headache, heart burn, diarrhea, tingling, and in some instances burning of the tongue and mouth. Melatonin was the least well-tolerated with seven out of ten studies reporting some adverse effects, the most common being headache, nausea, and diarrhea.

The majority of studies did not report their sources of funding; therefore, it was difficult to assess conflicts of interest. Five studies reported some affiliations with industry; one indicating involvement with supply, design, and publication; one for the supply of materials; one on the recruitment of participants; and two were unclear on its impact on published results. This may pose a risk of bias that might be favored towards beneficial effects of these compounds [46]. Five studies indicated they received funding sources with low risk of conflict of interest.

One of the major limitations of our review is the limited number of high-quality studies free from a high risk of bias and with adequate sample size to demonstrate effects. The studies included in the meta-analysis mostly had a small number of participants and were of poor quality as rated by the Cochrane risk of bias tools. As such the GRADE approach to make recommendations was abandoned (despite initially intended). The study protocols were often poorly reported with respect to duration, direction, and type of flight test (computer simulations and air flights). As a result, no subanalysis of the effects of flight duration on the efficacy of melatonin for jetlag could be conducted. As the body of evidence grows, further examination of the impacts of these varied conditions may be assessed in a manner not currently possible.

To our knowledge this is the first extensive review examining the efficacy of functional foods, beverages, and supplements that claim to alleviate symptoms experienced in air flight. The systematic review was limited to studies published in English and may have missed studies in other languages. However, the search strategy employed was comprehensive (spanning 674 lines in Medline via Ovid) conducted across multiple electronic databases.

## 5. Conclusions

Overall, from the range of functional foods, beverages, and supplements identified in the scoping review, there is limited research performed within flight or simulation settings to assess claims made. Of the studies available, Pycnogenol and melatonin may have beneficial effects on edema and jetlag, respectively. However, due to the poor quality and small number of studies, no recommendation for the use of these products can be made until more research emerges.

## Figures and Tables

**Table 1:** Health claims made by products (*n* = 111) identified in scoping review of functional foods, beverages and nutritional supplements that target air travel symptoms.

Health Claim Category ^1^	*n* ^2^	Percentage of Products
Fatigue	42	37.8
Immunity	41	36.9
Jetlag	36	32.4
Sleep	36	32.4
Hydration Status	31	27.9
Anxiety	29	26.1
Cardiovascular	24	21.6
Cognitive Ability	16	14.4
Gastrointestinal Symptoms	16	14.4
Radiation/Oxidative Stress	15	13.5
Nausea	11	9.9
Inflammation	5	4.5

^1^ Health claims are classified according to statements made by the functional foods, beverages, and nutritional supplements rather than clinical presentations; ^2^ Products may make more than one claim; therefore, the cumulative sum does not add to total number of products (*n* = 111).

**Table 2:** Ingredients advertised by products (*n* = 111) identified in scoping review of functional foods, beverages, and nutritional supplements that target air travel symptoms.

Ingredient	Product (*n*) ^1^	Percentage of Products Containing
**Vitamins**	44	39.6
A	0	0.0
B (not further defined)	16	14.4
B1	8	7.2
B2	7	6.3
B3	7	6.3
B5	5	4.5
B6	14	12.6
B7	3	2.7
B9	3	2.7
B12	8	7.2
C	22	19.8
D	4	3.6
E	4	3.6
**Minerals**	41	36.9
Electrolytes (not further defined)	13	11.7
Sodium	6	5.4
Potassium	7	6.3
Calcium	3	2.7
Magnesium	19	17.1
Chloride	4	3.6
Bicarbonate	1	0.9
Zinc	20	18.0
Other	18	16.2
**Macronutrients**	35	31.5
Glucose/Sugar/Carbohydrate	5	4.5
Amino Acids/Protein	26	23.4
Dietary Fiber	2	1.8
**Pharmacological**	16	14.4
Caffeine	3	2.7
Melatonin	14	12.6
**Herbal/Supplement**	74	66.7
Pycnogenol	7	6.3
Other	72	64.9

^1^ Products may advertise more than one ingredient in each category; therefore, the cumulative sum does not add to the total number of products (*n* = 111).

**Table 4:** Summary of key results of included studies conducted within (**a**) flight settings and (**b**) simulated flight settings.

(a)						
Author, Year, Citation	Agent	Key Outcome and Measurement Method(s) ^1^	Key Results ^1^	Adverse Effects ^1^	Funding and Conflicts of Interest ^1^	Overall Risk of Bias ^2^
Cesarone et al., 2001 [20]	*Centella asiatica*	Edema: subjective analogue scale line before and after flight Rate of ankle swelling method: NR	Edema: supplementation was associated with reduced edema after 9 h of flight (I = 2.6, C = 3.6, *p* < 0.05) when compared to control Rate of Ankle Swelling: supplementation reduced rate of swelling after 3 h of flight (I = 1.2, C = 1.7, *p* < 0.05) when compared to control	None	Funding: NR	High
Tiralongo et al., 2016 [21]	Elderberry	Cold diagnosis and length: Jackson score (daily)	Cold diagnosis: NS difference between number of participants diagnosed with colds (I = 12, C = 17, *p* = 0.2). Placebo group had longer collective cold episode in days (I = 57, C = 117, *p* = 0.05) and higher symptom score (I = 247, C = 583, *p* = 0.02) than intervention.	*n* = 5–cold like symptoms, fatigue and kidney pain	Industry provided capsules, partial involvement in study design and results publication	High
Tiralongo et al., 2012 [22]	*Echinacea*	Quality of Life: Wisconsin Upper Respiratory Symptom Survey (WURSS-44) at 14 days prior to travel, <1 week and 4 weeks after return flight Respiratory disorder symptom score (RDS+): A WURSS-44 score of 17+ at same time points	Quality of Life: Placebo group had a higher median WURSS-44 score than *Echinacea* group (I = 13, C = 26, *p* = 0.05) at within 1 week return time point. NS for baseline (14 days prior) and follow-up (4 weeks post) RDS+: Percentage of participants reporting respiratory illness (WURSS-44 > 17) was lower in *Echinacea* group than placebo (I = 43%, C = 57%, *p* = 0.05) at 1 week return time point and 4 week time point (I = 25%, C = 39%, *p* = 0.03). Baseline NS.	*n* = 3: vomiting, headache, heart burn, diarrhea *n* = 2, tingling, burning of tongue and mouth	Industry funding leveraged from an AusIndustry grant through Australian Government	High
Arendt et al., 1988 [23]	Melatonin	Jetlag: self-reported using 10 cm visual analogue scale on 6–7 days post flight.	Jetlag (eastbound): Melatonin improved self-reported jetlag ratings compared to placebo (mean (SD): I = 21.4 (19.4 *), C = 39.2 (30.7 *), *p* = 0.01015) Jetlag (westbound): Melatonin improved self-reported jetlag ratings compared to placebo (mean (SD): 19.9 (20.1 *), C = 44.5 (30.1 *), *p* = 0.00136 * SD back calculated as per Cochrane handbook [16]	*n* = 6 Headache *n* = 5 Nausea *n* = 4 worsened symptoms	Funding: NR	Some Concerns
Nickelsen et al., 1991 [24]	Melatonin	Jetlag: self-reported using visual analogue scale daily and overall retrospective rating.	Jetlag (eastbound): NS in overall self-reported jetlag between melatonin and placebo group (mean (SD): I = 5.2 (2.5), C = 6.6 (2.1), *p* = 0.071) Jetlag (westbound): NS in overall self-reported jetlag between melatonin and placebo group (mean (SD): I = 2.6 (1.8), C = 3.5 (2.6), *p* = 0.214)	NR	Funding: NR	Serious
Petrie et al., 1989 [25]	Melatonin	Jetlag: self-reported using visual analogue scale on arrival and 16:00 days 1–5, 7 and 10	Jetlag (both east- and westbound): Melatonin group reported less jetlag than placebo on day 10 (mean (SD): I = 2.15 (0.99), C = 3.40 (1.47), *p* < 0.01)	NR	Funding: NR	Some concerns
Petrie et al., 1993 [26]	Melatonin	Jetlag: self-reported using visual analogue scale daily at 16:00 h for 6 days and day 6 retrospective rating	Jetlag (westbound): Early melatonin group had higher retrospective rating of jetlag on day 6 than late melatonin (mean (SD): I_1 5.0 mg_ = 66.7 (22.7), I_2 0.5 mg_ = 37.7 (28.0), *p* < 0.05) but similar to placebo group (mean (SD): C = 64.7 (24.3), *p* > 0.05)	*n* = 5 for early melatonin: sleeping difficulties, drowsiness, headaches and depression	Funding: NR	High
Arendt et al., 1987 [27]	Melatonin	Jetlag: self-reported using 10 cm visual analogue scale on day 7 after arrival	Jetlag (eastbound): melatonin group reported less jetlag than placebo group (mean (SD): I = 11.3 (9.3) *, C = 55.2 (38.2) *, *p* < 0.01 * values from previous systematic review on melatonin [2]	NR	Funding: Horner Ltd./Nabisco Airline and hotel supplied flights and accommodation	High
Claustrat et al., 1992 [28]	Melatonin	Treatment efficiency of melatonin on jetlag: self-reported on day 8 after arrival (10 cm visual analogue scale).	Treatment efficiency (eastbound): melatonin had a greater treatment efficiency score (median: I = 73, C = 48, *p* < 0.05) than placebo group. * values from previous systematic review on melatonin: mean (SD): I = 34.5 (30.9), C = 52.8 (36.2) [2]	*n* = 2 hypnotic effects *n* = 1 tachycardia *n* = 2 heavy head	Funding: DRET grant	Serious
Edwards et al., 2000 [29]	Melatonin	Jetlag: self-reported using visual analog scale (range 1–10) and Liverpool Jetlag Questionnaire (07:00 ± 08:00 h, 12:00 ± 13:00 h, 16:00 ± 17:00 h and 19:00 ± 20:00 h over 6 days)	Jetlag (eastbound): NS in subjective ratings of jetlag between melatonin and placebo groups over 6 days (*p* = 0.741) and day 6 time point (*p* = 0.833)	*n* = 6 headache, *n* = 4 dizziness, *n* = 6 “rocking” (*n* = 5 melatonin *p* = 0.036)	Funding: NR	High
Spitzer et al., 1999 [30]	Melatonin	Jetlag: Columbia Jetlag Scale daily over 7 days	Jetlag (eastbound): NS in ratings of jetlag between melatonin and placebo groups (*p* = 0.62)	*n* = 1 difficulty swallowing and breathing	Funding: New York State Office of Mental Health. Recruitment: pharmaceutical sponsored education program.	High
Suhner et al., 2001 [31]	Melatonin	Jetlag: Scale (range: 1–3) every evening and 100 mm visual analog scale on day 4 Treatment effectiveness: 100 mm visual analog scale on day 4	Jetlag (eastbound): NS in subjective ratings between melatonin and placebo groups (*p* > 0.05) Treatment effectiveness (eastbound): Melatonin more effective than placebo (mean (SEM): I_1_ = 41.1 (4.9), C = 25.1 (4.4) *p* < 0.05). * values interpreted from figure. This study was excluded from meta-analysis	*n* = 17 including: diarrhea, fever, nausea, headache	Funding: NR	High
Suhner et al., 1998 [32]	Melatonin	Jetlag: symptoms questionnaire every evening on a 3-point scale	Jetlag (eastbound): NS in ratings of jetlag between melatonin and placebo group (*p* > 0.05)	Some–authors attributed to jetlag	Funding: NR	High
Cesarone et al., 2003 [33]	Pinokinase	Edema: score based on parametric data (edema tester, variations in ankle circumference, volume measurements) and subjective assessment of swelling and discomfort on an analogue scale line (range: 0–10) DVT: ultrasound scan of venous system	Edema: lower edema score after flight in Pinokinase group than control group (mean (SD): I = 7.54 (0.8), C = 9.8 (0.5), *p* < 0.05) DVT: reduced incidence of DVT in Pinokinase group than control group (*n*: I = 0, C = 5, *p* < 0.025)	NR	Funding: not sponsored by company producing materials quoted	High
Belcaro et al., 2018 [34]	Pycnogenol	Edema: score based on parametric data (edema tester, variations in ankle circumference, volume measurements) and subjective assessment of swelling and discomfort on an analogue scale line DVT: ultrasound scan of venous system >24 h before flight and >30 h return flight	Edema (low risk group): Pycnogenol group had lower edema than standard management (C_1_) and compression stockings (C_2_): mean (SD): I = 1.03 (0.2), C_1_ = 2.4 (0.2), C_2_ = 2.1 (0.3), *p* < 0.05 Edema (moderate risk group): Pycnogenol group had lower edema than standard management (C_1_) and stockings (C_2_): mean (SD): I = 1.7 (0.3), C_1_ = 3.6 (0.2), C_2_ = 3.4 (0.2), *p* < 0.05 Edema (high risk group): Pycnogenol group had lower edema than standard management and compression stockings: mean (SD): I = 1.6 (0.4), C_1_ = 3.4 (0.2), C_2_ = 3.4 (0.2), *p* < 0.05 DVT (low risk): Incidence: I = 0, C_1_ = 0, C_2_ = 0, nil *p*-value DVT (moderate risk): Incidence: I = 0, C_1_ = 1, C_2_ = 0, nil *p*-value DVT (high risk): Incidence: I = 0, C_1_ = 1, C_2_ = 0, nil *p*-value	NR	Funding: not sponsored by company producing materials quoted	Serious
Belcaro et al., 2008 [35]	Pycnogenol	Study 1: Jetlag: self-reported using visual analog scale (range 1–10) < 48 h post flight	Jetlag: duration (hours) of signs/symptoms of jetlag were reduced in Pycnogenol group when compared to controls (mean (SD): I = 12.2 (7), C = 39.3 (0.8), *p* < 0.05).	NR	Funding: Italian Society for Vascular Investigations (ISVI), Ministry of Scientific Research (MURST) and Department of Biomedical Sciences, G’D’Annunzio University	Serious
		Study 2: Edema: CT scan of brain < 28 h post flight and evaluated using cerebral CT edema scale (range: 0–5)	Edema: lower edema score in Pycnogenol group than control group (mean (SD): I = 1.2 (4.0), C = 3.2 (4.0), *p* < 0.05) * SD back calculated as per Cochrane handbook [16]			
Belcaro et al., 2004, RCT [36]	Pycnogenol	DVT/SVT: ultrasound scan < 90 min before flight and <2 h post flight	DVT: Pycnogenol group had a lower incidence than control group (I = 0, C = 1, nil *p*-value) SVT: Pycnogenol group had a lower incidence than control group (I = 0, C = 4, *p* < 0.05)	NR	Funding: not sponsored by company producing materials quoted	High
Cesarone et al., 2005 [37]	Pycnogenol	Edema: edema score (0–12) composed of: analogue line by measuring observer, edema perceived by participant, edema perceived by observer and associated edema signs or symptoms	Edema: The increase in edema score of Pycnogenol group was less than controls following flight (mean (SD) I = 3.6 (9.3), C = 8.2 (9.3), *p* < 0.05) * SD back calculated as per Cochrane handbook [16]	NR	Funding: NR Materials supplied by Pycnogenol company without conditions	Serious
**(b)**						
**Author, Year, Citation**	**Agent**	**Key Outcome and Measurement Method(s) ^1^**	**Key Results ^1^**	**Adverse Effects ^1^**	**Funding and Conflicts of Interest ^1^**	**Overall Risk of Bias ^2^**
Caska et al., 2007 [38]	Caffeine	Flight performance: horizontal and vertical deviations from prescribed flight path at baseline and 30 min post intervention	Flight performance: NS difference between groups in both mean horizontal (*p* = 0.60) and vertical deviations (*p* = 0.77)	NR	Funding: NR	High
Dagan et al., 2006 [39]	Caffeine	Flight performance: deviations from prescribed altitude and velocity at 23:00, 01:00, 03:00, 05:00, 07:00, 09:00, and 11:00 h.	Flight performance: Caffeine decreased deviations from altitude from baseline (mean difference = −191.1, *p* = 0.0093) at 03:00 and velocity from baseline (mean difference: −11.2, *p* = 0.0115) and control (mean difference: −8.8, *p* = 0.0444) at 05:00 compared within participants	NR	Funding: NR	Some concerns
Lindseth et al., 2013 [40]	Fluid	Flight performance: deviations from prescribed airspeed control, heading control, and altitude control.	Flight performance: NS within subject scores between fluid diet (mean (SD): I _high fluid_ = 231,600.5 (315,627.7), C _low fluid_ = 278,986.8 (194,077.3), *p* = 0.97) compared within participants. Subgroup analysis: flight performance of individuals that were dehydrated (1–3% participant weight loss) and on a low fluid diet was poorer than those without dehydration (mean (SD): 1–3% body weight loss = 449,005.2 (43,909.0), no weight loss 193,234.9 (72,055.9), *p* = 0.002)	NR	Funding: US Army Biomedical Research Command and National Institutes of Health	Some concerns
Hinninghofen, et al., 2006 [4]	Fiber	Gastric emptying: ^13^CO_2_ breath samples-% difference from baseline per minute and cumulatively over 4 h Symptom: Score (range: 1–5) of abdominal pain, distension, bloating, belching, heart burn, and general wellbeing	Gastric emptying: delayed at 2500 m altitude on a high fiber when compared to low fiber (mean (SD): I _high fiber_ 146.31 (58.41) min, C _low fiber_ 193.91 (54.34) min, *p* = 0.039) Symptoms: high reports of distention (mean (SD): I _high fiber_ 1.33 (0.3), C _low fiber_ 1.07 (0.15),0 *p* = 0.022) and bloating (mean: I _high fiber_ 1.82 (0.47), C _low fiber_ 1.34 (0.35), *p* = 0.016) at 2500 m altitude on a high fiber when compared to low fiber	High dietary fiber at 2500 altitude may increase gastrointestinal symptoms	Funding: NR	High
Lindseth et al., 2011 [41]	Macro-nutrients	Flight performance: deviations from prescribed airspeed control, heading control, and altitude control	Flight performance: I_1 high carbohydrate,_ I_3 high fat_ and C diets made fewer errors than I_2 high protein_ diet group (mean (SD): I_1_ = 206.1 (97.6), I_2_ 250.9 (109.8), I_3_ = 198.2 (100.3), C = 217.5 (135.9), *p* = 0.05) compared within participants.	NR	Funding: U.S. Army Biomedical Research Award and the National Institutes of Health	Some concerns

^1^ Abbreviations used: not significant (NS), not reported (NR), intervention group (I), control/comparator group (C), deep vein thrombosis (DVT) and superficial venous thrombosis (SVT). ^2^ Cochrane risk of bias tool Rob-2 assesses risk of bias as: high, some concerns and low and the Robins-I tool assesses risk of bias as: serious, low, and no information.

## Data Availability

The majority of the data is already supplied in the Supplementary Files. If additional information is required, please contact the corresponding author.

## Supplementary Materials

The following are available online at https://www.mdpi.com/2072-6643/13/3/961/s1, Table S1. Electronic database search strategy: Medline (via ovid) for scoping review of functional foods, beverages and supplements for flight related symptoms, Table S2. Search engine search strategy for scoping review of functional foods, beverages and supplements for flight related symptoms, Table S3. Full text articles excluded (*n* = 43) with reasons, Table S4. Electronic database search strategy: Medline (via Ovid) for systematic review.
