# Supplementary material for: Efficacy of Functional Foods, Beverages, and Supplements Claiming to Alleviate Air Travel Symptoms: Systematic Review and Meta-Analysis
Source: Nutrients. 2021 Mar 16;13(3):961. doi: 10.3390/nu13030961 (PMC8002180; doi:10.3390/nu13030961)
Supplement: Supplementary file 1 [file nutrients-13-00961-s001.pdf]

nutrients MDPI

Table S1. Electronic database search strategy: Medline (via ovid) for scoping review of functional foods, beverages and supplements for flight related symptoms.

Search No.	Search Statement	No. of Citations Retrieved
1	exp Beverages/ or exp Dietary supplements/ or Food, fortified/ or Functional food/ or drink*.tw. or food.tw. or snack*.tw. or diet*.tw. or nutrition.tw. or supplement.tw.	1 221 282
2	((Air adj2 (passenger* or transport* or travel*)) or ((Air or cabin or civil or commercial) adj5 (pilot* or attendant* or steward* or hostess or crew*)) or ((Flight or flights or flying) not (spectro* or TOF or time-of-flight)) or airplane* or aviation).tw.	40 925
3	exp Jet Lag Syndrome/ or exp venous thrombosis/ or exp Sleep/ or exp dehydration	147 558
4	2 or 3	187 648
5	1 and 4	7 548
6	Limit 6 to ("all adult (19 plus years)" and english)	2 453

Table S2. Search engine search strategy for scoping review of functional foods, beverages and supplements for flight related symptoms.

Search No.	Search Statement
1	Drinks or beverages for air flight
2	Drinks or beverages for jet lag
3	Drinks or beverages for deep vein thrombosis
4	Drinks or beverages for sleeping on airplanes
5	Food for air flight
6	Food for jet lag
7	Food for deep vein thrombosis
8	Food for sleeping on airplanes
9	Snacks for air flight
10	Snacks for jet lag
11	Snacks for deep vein thrombosis
12	Snacks for sleeping on airplanes
13	Supplements for air flight
14	Supplements for jet lag
15	Supplements for deep vein thrombosis
16	Supplements for sleeping on airplanes
17	Hydration on airplanes

Table S3. Full text articles excluded (n = 43) with reasons.

Reason	Article
Inappropriate Population	• Beaumont M.; Batejat D.; Pierard C.; Van Beers P.; Denis J. B.; Coste O.; Doireau P.; Chauffard F.; French J.; Lagarde D. Caffeine or melatonin effects on sleep and sleepiness after rapid eastward transmeridian travel. *J Appl Physiol* **2004**, *96*, 50-58. doi:http://dx.doi.org/10.1152/japplphysiol.00940.2002<br>• Kilpelainen A. A.; Huttunen K. H.; Lohi J. J.; Lyytinen H. Effect of caffeine on vigilance and cognitive performance during extended wakefulness. *The International Journal of Aviation Psychology* **2010**, *20*, 144-159. doi:http://dx.doi.org/10.1080/10508411003617847<br>• Lagarde D.; Chappuis B.; Billaud P. F.; Ramont L.; Chauffard F.; French J. Evaluation of pharmacological aids on physical performance after a transmeridian flight. *Med Sci Sports Exerc* **2001**, *33*, 628-634. doi:http://dx.doi.org/10.1097/00005768-200104000-00018<br>• Lucertini M.; Mirante N.; Casagrande M.; Trivelloni P.; Lugli V. The effect of cinnarizine and cocculus indicus on simulator sickness. *Physiol Behav* **2007**, *91*, 180-190. doi:http://dx.doi.org/10.1016/j.physbeh.2007.02.008<br>• Paul M. A.; Brown G.; Buguet A.; Gray G.; Pigeau R. A.; Weinberg H.; Radomski M. Melatonin and zopiclone as pharmacologic aids to facilitate crew rest. *Aviat Space Environ Med* **2001**, *72*, 974-984<br>• Pierard C.; Beaumont M.; Enslen M.; Chauffard F.; Tan D. X.; Reiter R. J.; Fontan A.; French J.; Coste O.; Lagarde D. Resynchronization of hormonal rhythms after an eastbound flight in humans: Effects of slow-release caffeine and melatonin. *Eur J Appl Physiol* **2001**, *85*, 144-150. doi:http://dx.doi.org/10.1007/s004210100418<br>• Paul M. A.; Gray G.; Sardana T. M.; Pigeau R. A. Melatonin and zopiclone as facilitators of early circadian sleep in operational air transport crews. *Aviat Space Environ Med* **2004**, *75*, 439-443<br>• Stokes A. F.; Belger A.; Banich M. T.; Taylor H. Effects of acute aspartame and acute alcohol ingestion upon the cognitive performance of pilots. *Aviat Space Environ Med* **1991**, *62*, 648-653<br>• Zhang L. L.; Liu H. Q.; Yu X. H.; Zhang Y.; Tian J. S.; Song X. R.; Han B.; Liu A. J. The Combination of Scopolamine and Psychostimulants for the Prevention of Severe Motion Sickness. *CNS Neuroscience and Therapeutics* **2016**. doi:http://dx.doi.org/10.1111/cns.12566
Inappropriate Intervention	• Cesarone M. R.; Belcaro G.; Geroulakos G.; Griffin M.; Ricci A.; Brandolini R.; Pellegrini L.; Dugall M.; Ippolito E.; Candiani C.; Simeone E.; Errichi B. M.; Di Renzo A. Flight microangiopathy on long-haul flights: prevention of edema and microcirculation alterations with Venoruton. *Clin Appl Thromb Hemost* **2003**, *9*, 109-114<br>• Cesarone M. R.; Belcaro G.; Nicolaides A. N.; Incandela L.; De Sanctis M. T.; Geroulakos G.; Lennox A.; Myers K. A.; Moia M.; Ippolito E.; Winford M. Venous thrombosis from air travel: The LONFLIT3 study: Prevention with aspirin vs low-molecular-weight heparin (LMWH) in high-risk subjects: A randomized trial. *Angiology* **2002**, *53*, 1-6<br>• Paul M. A.; Gray G.; MacLellan M.; Pigeau R. A. Sleep-inducing pharmaceuticals: A comparison of melatonin, zaleplon, zopiclone, and temazepam. *Aviat Space Environ Med* **2004**, *75*, 512-519<br>• Young R.; Molesworth B. R. The effects of caffeine on learning: A pilot's perspective. *International Journal of Applied Aviation Studies* **2011**, *11*, 85-95<br>• van Drongelen A.; Boot C. R.; Hlobil H.; Twisk J. W.; Smid T.; van der Beek A. J. Evaluation of an mHealth intervention aiming to improve health-related behavior and sleep and reduce fatigue among airline pilots. *Scand J Work Environ Health* **2014**, *40*, 557-568. doi:https://dx.doi.org/10.5271/sjweh.3447

Table S3. (Continued)

Reason	Article
Inappropriate Outcome	• Barattini P.; Dolci C.; Montaruli A.; Roveda E.; Carandente F. Resynchronization of blood pressure circadian rhythm after westward trans-7-meridian flight with and without melatonin treatment. *Aviation, Space, and Environmental Medicine* **2001**, *72*, 221-224<br>• Deixelberger-Fritz D.; Tischler M.; Kallus K. Changes in Performance, Mood State and Workload Due to Energy Drinks in Pilots. *International Journal of Applied Aviation Studies* **2003**, *3*, 195-205<br>• Molesworth B. R. C.; Young R. Caffeine: Mitigating the Effects of Fatigue on the Flight Deck? *Proceedings of the Human Factors and Ergonomics Society Annual Meeting* **2008**, *52*, 1-5. doi:10.1177/154193120805200102<br>• Stokes A. F.; Belger A.; Banich M. T.; Bernadine E. Effects of alcohol and chronic aspartame ingestion upon performance in aviation relevant cognitive tasks. *Aviat Space Environ Med* **1994**, *65*, 7-15<br>• Takahashi T.; Sasaki M.; Itoh H.; Ozone M.; Yamadera W.; Hayshida K.; Ushijima S.; Matsunaga N.; Obuchi K.; Sano H. Effect of 3 mg melatonin on jet lag syndrome in an 8-h eastward flight. *Psychiatry Clin Neurosci* **2000**, *54*, 377-378. doi:10.1046/j.1440-1819.2000.00722.x<br>• Takahashi T.; Sasaki M.; Itoh H.; Yamadera W.; Ozone M.; Obuchi K.; Hayashida K.-i.; Matsunaga N.; Sano H. Melatonin alleviates jet lag symptoms caused by an 11-hour eastward flight. *Psychiatry Clin Neurosci* **2002**, *56*, 301-302. doi:http://dx.doi.org/10.1046/j.1440-1819.2002.00988.x
Inappropriate Study Design	• Manfredini R.; Manfredini F.; Conconi F. Standard melatonin intake and circadian rhythms of elite athletes after a transmeridian flight. *J Int Med Res* **2000**, *28*, 182-186. doi:http://dx.doi.org/10.1177/147323000002800403<br>• Cardinali D. P.; Bortman G. P.; Liotta G.; Perez Lloret S.; Albornoz L. E.; Cutrera R. A.; Batista J.; Ortega Gallo P. A multifactorial approach employing melatonin to accelerate resynchronization of sleep-wake cycle after a 12 time-zone westerly transmeridian flight in elite soccer athletes. *J Pineal Res* **2002**, *32*, 41-46. doi:10.1034/j.1600-079x.2002.10820.x<br>• Cardinali D. P.; Furio A. M.; Reyes M. P.; Brusco L. I. The use of chronobiotics in the resynchronization of the sleep-wake cycle. *Cancer Causes Control* **2006**, *17*, 601-609. doi:http://dx.doi.org/10.1007/s10552-005-9009-2<br>• Cotter J. D.; Parr E. B.; Silcock P.; Nyhof F.; Rehrer N. J. Physiological testing of a beverage system designed for long-haul air travel. *Extreme Physiology & Medicine* **2015**, *4*, A61. doi:10.1186/2046-7648-4-S1-A61<br>• Hamada K.; Doi T.; Sakurai M.; Matsumoto K.; Yanagisawa K.; Suzuki T.; Kikuchi N.; Okuda J.; Miyazaki H.; Okoshi H.; Zeniya M.; Asukata I. Effects of hydration on fluid balance and lower-extremity blood viscosity during long airplane flights. *J Am Med Assoc* **2002**, *287*, 844-845<br>• Scurr J. H.; Gulati O. P. Zinopin® – the rationale for its use as a food supplement in traveller's thrombosis and motion sickness. *Phytother Res* **2004**, *18*, 687-695. doi:10.1002/ptr.1575<br>• Vats P.; Singh V. K.; Singh S. N.; Singh S. B. Glutathione metabolism under high-altitude stress and effect of antioxidant supplementation. *Aviat Space Environ Med* **2008**, *79*, 1106-1111. doi:http://dx.doi.org/10.3357/ASEM.2305.2008<br>• Actrn. A randomised controlled trial to evaluate the effectiveness of ACCESS (Airline Cabin Crew Educational Strategies for Sleep) program for tired airline cabin crew, *http://www.who.int/trialsearch/Trial2.aspx?TrialID=ACTRN12620000394943* **2020**<br>• Wilson D.; Driller M.; Johnston B.; Gill N. The effectiveness of a 17-week lifestyle intervention on health behaviors among airline pilots during COVID-19, *Journal of Sport & Health Science*, **2020**, 20, 1-20

Table S3. (Continued)

Reason	Article
Non-English Text	• Birkmayer G. D.; Kay G. G.; Viirre E. Stabilized NADH as a counter measure for jet lag. [German] (Stabilisiertes NADH (ENADA) verbessert die durch jetlag reduzierte hirnleistung.). *Wien Med Wochenschr* **2002**, *152*, 450-454<br>• Birkmayer G. D.; Kay G. G.; Vurre E. [Stabilized NADH (ENADA) improves jet lag-induced cognitive performance deficit]. *Wien Med Wochenschr* **2002**, *152*,450-454<br>• Luo X. M.; Hu Y. H.; Yu J.; Wang H.; Xu Q. Y.; Zhan H. [Effects of tea polyphenols on microcirculation and antioxidation in aircrew]. *Hangtian Yixue Yu Yixue Gongcheng/Space Medicine & Medical Engineering* **1999**, *12*, 338-341<br>• Morell T. Combat of fatigue by active substances. *Dtsch Med Wochenschr* **1940**, *66*, 398. doi:http://dx.doi.org/10.1055/s-0028-1121534<br>• Okuda J.; Hamada K.; Doi T.; Sakurai M.; Kikuchi N.; Zeniya M.; Asukata I. Effect of a carbohydrate-electrolyte beverage on serum and urine osmolality during long flights. *Japanese Journal of Aerospace and Environmental Medicine* **2003**, *40*, 117-124<br>• Tishchenko A. A.; Rudko E. A.; Markovich D. Y.; Malinin A. V.; Vasiliev K. Y. Transmeridian Jet Lag and Its Pharmacological Correction. *Aviakosm Ekolog Med* **2016**, *50*, 63-68
Full text not available and Clinical Trial Registers	• King C.; Bickerman H.; Bouvet W.; Harrer C.; Oyler J.; Seitz C. Aviation nutrition studies: I. Effects of pre-flight and in-flight meals of varying composition with respect to carbohydrate, protein and fat. *J Aviat Med* **1945**, *16*, 69-84<br>• Grant F. W.; Smith R. F. Psycho-motor performance related to vitamin intake. *J Am Diet Assoc* **1952**, *28*, 1046-1049<br>• Greenleaf J. E.; Farrell P. A.; Loomis J. L.; Fedele M. J.; West J.; Rossler A.; Hinghofer-Szalkay H. Sodium chloride-citrate beverages attenuate hypovolemia in men resting 12 h at 2800 m altitude. *Aviat Space Environ Med* **1998**, *69*, 936-943<br>• Actrn Investigating the effectiveness of Elderberry capsules in the prevention of upper respiratory symptoms associated with long distance travel. *http://wwwwhoint/trialsearch/Trial2aspx?TrialID=ACTRN12612001301853* **2012**<br>• Nct Effects of Hydrocortisone, Melatonin, and Placebo on Jet Lag. *Https://clinicaltrialsgov/show/nct00097474* **2004**
Abstract Only	• D'Oliveira T. C. Markers of circadian disturbances in cabin crew: Combining cortisol and melatonin responses with self-reporting measures. *Brain Behav Immun* **2016**, *57 (Supplement 1)*, e13-e14. doi:http://dx.doi.org/10.1016/j.bbi.2016.07.047<br>• Tiralongo E.; Lea R.; Wee S.; Hanna M.; Griffiths L. The effectiveness of a standardised Echinacea preparation in preventing colds, flus and other respiratory disorders for air-travellers. *Planta Medica Conference: 7th Tannin Conference* **2010**, *76*. doi:http://dx.doi.org/10.1055/s-0030-1264287<br>• Tiralongo E.; Wee S.; Lea R. A. Does elderberry benefit intercontinental air travellers? *Advances in Integrative Medicine* **2015**, *2*, 117. doi:http://dx.doi.org/10.1016/j.aimed.2015.07.008

Table S4. Electronic database search strategy: Medline (via ovid) for systematic review.

Group	Search	Query	Records Retrieved[1]
Product delivery or consumption method	1	exp Beverages/	131806
	2	exp Candy/	3749
	3	exp Dietary supplements/	70813
	4	exp Fermented foods/	26771
	5	exp Tablets/	23960
	6	Capsules/	12370
	7	Food, fortified/	9080
	8	Functional food/	1675
	9	Plant extracts/	108127
	10	Plants, medicinal/	58475
	11	Powders/	13357
	12	Rehydration solutions/	1432
	13	Wakefulness-promoting agents/	189
	14	((Alert* or stimulat* or wake*) adj3 (agent* or beverage* or compound* or drink*)).tw.	10072
	15	((Electrolyte or rehydrat* or sport*) adj3 (drink* or beverage* or solution*)).tw.	7519
	16	((Ferment* or fortif* or functional or health*) adj5 (bar* or beverage* or biscuit* or cand*3 or confectionary or cookie* or drink* or food* or meal* or snack*)).tw.	60951
	17	(Herbal adj3 (tea* or supplement* or extract*)).tw.	4899
	18	(Medicinal adj3 (herb* or plant* or tea* or supplement*)).tw.	22798
	19	(Supplement adj1 (food or nutrition* or diet*)).tw.	8102
	20	Capsule*.tw.	75069
	21	Chewing gum.tw.	1748
	22	Eugeroic.tw.	1
	23	Plant extract*.tw.	9503
	24	Powder*.tw.	69177
	25	Superfood*.tw.	50
	26	Tablet*.tw.	50604
	27	or/1-26	640084
Other	28	exp Bifidobacterium/	5642
	29	exp Chlorella/	4252
	30	exp Lactobacillus/	27445
	31	exp Mentha/	935
	32	exp Moringa/	593
	33	exp Prunus/	4122
	34	exp Sambucus/	339
	35	Acacia/	1164
	36	Actinidia/	782
	37	Agaricus/	1152
	38	Albizzia/	195
	39	Aloe/	1307
	40	Althaea/	59
	41	Ananas/	486
	42	Andrographis/	424
	43	Arnica/	201

	44	Aspalathus/	150
	45	Astragalus propinquus/	658
	46	Avena/	2208
	47	Bambusa/	388
	48	Beta vulgaris/	1928
	49	Blueberry plants/	944
	50	Brassica rapa/	1722
	51	Brassica/	6753
	52	Bromelains/	1344
	53	Caffeine/	22850
	54	Capsicum/	3119
	55	Carica/	846
	56	Chamomile/	407
	57	Charcoal/	10901
	58	Chicory/	446
	59	Chlorophyll/	17791
	60	Chocolate/	306
	61	Choline/	18082
	62	Cinchona/	348
	63	Cinnamomum zeylanicum/	726
	64	Citrullus/	560
	65	Citrus sinensis/	1741
	66	Citrus/	6932
	67	Cocculus/	26
	68	Cacao/	3081
	69	Cocos/	1393
	70	Coffea/	1111
	71	Cordyceps/	881
	72	Corydalis/	306
	73	Curcuma/	1820
	74	Curcumin/	9597
	75	Cymbopogon/	377
	76	Cynara scolymus/	324
	77	Dioscorea/	842
	78	Echinacea/	753
	79	Eleutherococcus/	473
	80	Eschscholzia/	84
	81	Eucalyptus/	2367
	82	Euterpe/	140
	83	Ficus/	1051
	84	Foeniculum/	258
	85	Fucus/	356
	86	Fumaria/	48
	87	Garlic/	3377
	88	Gelsemium/	116
	89	Gentiana/	327
	90	Ginger/	1453
	91	Ginkgo biloba/	2759
	92	Glycyrrhiza/	2308
	93	Grape seed extract/	710
	94	Grifola/	214

	95	Hibiscus/	689
	96	Hordeum/	9246
	97	Humulus/	703
	98	Hyaluronic acid/	20576
	99	Hypericum/	2150
	100	Ilex paraguariensis/	301
	101	Kava/	480
	102	Lavandula/	434
	103	Leonurus/	181
	104	Lepidium/	279
	105	Litchi/	301
	106	Lycium/	553
	107	Malpighiaceae/	248
	108	Malus/	5031
	109	Marrubium/	71
	110	Matricaria/	246
	111	Melatonin/	18961
	112	Melissa/	203
	113	Menthol/	1857
	114	Milk thistle/	506
	115	Olea/	2898
	116	Opuntia/	474
	117	Oxygen/	161022
	118	Panax/	4856
	119	Papain/	6182
	120	Passiflora/	424
	121	Paullinia/	135
	122	Petrolatum/	1072
	123	Petroleum/	10836
	124	Peumus/	49
	125	Phyllanthus emblica/	261
	126	Pimpinella/	131
	127	Piper nigrum/	379
	128	Polyporus/	130
	129	Prebiotics/	2323
	130	Probiotics/	15696
	131	Propolis/	1963
	132	Punicaceae/	1251
	133	Quercetin/	8978
	134	Raphanus/	989
	135	Reishi/	969
	136	Resveratrol/	7985
	137	Rhodiola/	531
	138	Ribes/	381
	139	Rosa/	1148
	140	Rosmarinus/	732
	141	Rubus/	467
	142	Saccharomyces boulardii/	72
	143	Salix/	951
	144	Salvia officinalis/	321
	145	Schisandra/	745

	146	Scutellaria/	367
	147	Shiitake mushrooms/	571
	148	Strychnos nux-vomica/	130
	149	Syzygium/	815
	150	Taraxacum/	363
	151	Terminalia/	585
	152	Thioctic acid/	4007
	153	Tilia/	189
	154	Trametes/	702
	155	Turnera/	69
	156	Ubiquinone/	8929
	157	Urtica dioica/	275
	158	Vaccinium macrocarpon/	795
	159	Valerian/	705
	160	Vanilla/	136
	161	Verbascum/	79
	162	Verbena/	80
	163	Vitis/	8148
	164	Withania/	590
	165	Ziziphus/	542
	166	(Aloe adj2 (extract* or vera)).tw.	1449
	167	((California or californian) adj1 (poppy or poppies)).tw.	71
	168	(Cherr* adj1 (barbados or sweet or sour or tart)).tw.	755
	169	(Lime adj2 (blossom* or flower*)).tw.	20
	170	(Lemon adj2 (balm or verbena)).tw.	246
	171	Acacia.tw.	2520
	172	Acai.tw.	326
	173	Actinidia.tw.	688
	174	Agaricus blazei.tw.	263
	175	Albizzia.tw.	128
	176	Allium sativum.tw.	1416
	177	Aloysia citrodora.tw.	12
	178	Alpha lipoic acid.tw.	2575
	179	Althaea.tw.	88
	180	Amla.tw.	173
	181	Ananas comosus.tw.	308
	182	Andrographis.tw.	844
	183	Anise.tw.	382
	184	Aniseed.tw.	86
	185	Apple*.tw.	16061
	186	Arnica.tw.	386
	187	Artichoke.tw.	978
	188	Ashwagandha.tw.	280
	189	Aspalathus.tw.	156
	190	Astragalus.tw.	2530
	191	Avena sativa.tw.	1229
	192	Bamboo.tw.	3032
	193	Bambusa vulgaris.tw.	32
	194	Barley grass.tw.	39
	195	Beet.tw.	4756
	196	Beetroot*.tw.	611

	197	Bellis perennis.tw.	42
	198	Beta vulgaris.tw.	1463
	199	Bifidobacterium.tw.	7085
	200	BioPerine.tw.	6
	201	Black pepper.tw.	609
	202	Black raddish*.tw.	25
	203	Blackberr*.tw.	760
	204	Blackcurrant*.tw.	395
	205	Bladderwrack.tw.	12
	206	Blueberr*.tw.	2110
	207	Boldo.tw.	78
	208	Brassica.tw.	10347
	209	Broccoli.tw.	2133
	210	Bromelain*.tw.	1363
	211	Cacao.tw.	1037
	212	Caffein*.tw.	27923
	213	Camu Camu.tw.	44
	214	Capsicum.tw.	2822
	215	Caraway.tw.	257
	216	Carbo-vegetabilis.tw.	2
	217	Carica.tw.	1201
	218	Carob.tw.	369
	219	Carum carvi.tw.	115
	220	Cayenne.tw.	294
	221	Chaga mushroom*.tw.	28
	222	Chamomile.tw.	741
	223	Charcoal.tw.	10514
	224	Chicory.tw.	727
	225	Chilli.tw.	501
	226	China Officinalis.tw.	1
	227	Chlorella.tw.	5789
	228	Chlorophyll.tw.	26031
	229	Chocolate.tw.	4761
	230	Choline.tw.	37294
	231	Cinchona.tw.	1248
	232	Cinnamomum zeylanicum.tw.	261
	233	Cinnamon.tw.	2126
	234	Citrullus.tw.	665
	235	Citrus.tw.	10953
	236	Clove*.tw.	6250
	237	Cocculus.tw.	74
	238	Cocoa.tw.	2756
	239	Coconut.tw.	4077
	240	Cocos.tw.	862
	241	Coenzyme Q.tw.	2013
	242	Coenzyme Q10.tw.	3296
	243	Coffea crud*.tw.	6
	244	Coffee.tw.	13507
	245	Cordyceps.tw.	1424
	246	Corn silk.tw.	91
	247	Corydalis ambigua.tw.	18

	248	Cranberr*.tw.	1525
	249	Curcuma.tw.	2993
	250	Curcumin.tw.	12998
	251	Cymbopogon.tw.	613
	252	Cynara scolymus.tw.	201
	253	Daisy.tw.	551
	254	Damiana.tw.	36
	255	Dandelion.tw.	507
	256	Davidson plum*.tw.	0
	257	Dioscorea.tw.	1161
	258	Echinacea.tw.	1098
	259	Elderberr*.tw.	297
	260	Eleutherococcus senticosus.tw.	170
	261	Emblica officinalis.tw.	349
	262	Eschschol?zia californica.tw.	154
	263	Eucalyptus.tw.	3845
	264	Eugenia.tw.	614
	265	Euterpe.tw.	273
	266	Fennel.tw.	569
	267	Ficus.tw.	2076
	268	Fig.tw.	9688
	269	Fingerroot.tw.	20
	270	Foeniculum vulgare.tw.	381
	271	Fruitflow.tw.	4
	272	Fucus vesiculosis.tw.	7
	273	Fumaria.tw.	130
	274	Fumitory.tw.	16
	275	Ganoderma lucidum.tw.	1500
	276	Garam masala.tw.	7
	277	Garlic.tw.	5695
	278	Gelsemium.tw.	182
	279	Gentiana lutea.tw.	91
	280	Ginger.tw.	2825
	281	Ginkgo biloba.tw.	3394
	282	Ginseng.tw.	6984
	283	Glycyrrhiza glabra.tw.	630
	284	Goji berr*.tw.	108
	285	Grape*.tw.	17636
	286	Grifola.tw.	359
	287	Guarana.tw.	237
	288	Hericium erinaceus.tw.	201
	289	Hibiscus.tw.	1185
	290	Himematsutake.tw.	15
	291	Hops.tw.	1652
	292	Hordeum.tw.	4563
	293	Horehound lea*.tw.	1
	294	Hovenia dulcis.tw.	58
	295	Humulus lupulus.tw.	529
	296	Hyaluronic acid.tw.	17042
	297	Hypericum.tw.	2122
	298	Ilex paraguariensis.tw.	318

	299	Illawarra plum*.tw.	4
	300	Indian gooseberr*.tw.	55
	301	Inonotus obliquus.tw.	199
	302	Jamaican Dogwood.tw.	1
	303	John?s wort.tw.	1733
	304	Jujube.tw.	401
	305	Juvecol.tw.	0
	306	Kakadu plum*.tw.	7
	307	Kali phos.tw.	1
	308	Kava.tw.	648
	309	Kiwi*.tw.	1561
	310	Kombucha.tw.	130
	311	Lactobacillus.tw.	28001
	312	Lavandula.tw.	575
	313	Lavender.tw.	1033
	314	Lemon*.tw.	3986
	315	Lentinula edodes.tw.	482
	316	Leonurus cardiaca.tw.	56
	317	Lepidium.tw.	880
	318	Licorice.tw.	1861
	319	Lingzhi mushroom*.tw.	4
	320	Lion?s mane.tw.	51
	321	Liquorice.tw.	601
	322	Litchi.tw.	399
	323	Longan.tw.	254
	324	Lychee.tw.	152
	325	Lycium.tw.	857
	326	Maca.tw.	306
	327	Maitake.tw.	142
	328	Malpighiaceae.tw.	136
	329	Malus.tw.	1743
	330	Marrubium vulgare.tw.	87
	331	Marshmallow root.tw.	10
	332	Matricaria recutita.tw.	147
	333	Melatonin.tw.	23213
	334	Melissa officinalis.tw.	358
	335	Melon*.tw.	3087
	336	Mentha.tw.	1421
	337	Menthol.tw.	2683
	338	Meshimakobu.tw.	1
	339	Milk thistle.tw.	718
	340	Mint.tw.	1627
	341	Monk fruit.tw.	18
	342	Moringa.tw.	1051
	343	Motherwort.tw.	80
	344	Mountain pepper berr*.tw.	0
	345	Mullein lea*.tw.	1
	346	Nattokinase.tw.	139
	347	Nux vomica.tw.	213
	348	Oat*.tw.	14301
	349	Olea.tw.	1467

	350	Olive lea*.tw.	561
	351	Ophiocordyceps.tw.	254
	352	Opuntia.tw.	855
	353	Orange*.tw.	29657
	354	Oxygen.tw.	448541
	355	Panax.tw.	4493
	356	Papain.tw.	7899
	357	Papaya*.tw.	1997
	358	Passiflora.tw.	624
	359	Passion?fruit.tw.	326
	360	Passionflower.tw.	57
	361	Paullinia cupana.tw.	129
	362	Peach*.tw.	3805
	363	Peppermint.tw.	1133
	364	Petrolatum.tw.	1156
	365	Petroleum.tw.	12786
	366	Peumus.tw.	78
	367	Phellinus linteus.tw.	211
	368	Phosphoric acidum.tw.	0
	369	Phyllanthus emblica.tw.	246
	370	Pimpinella anisum.tw.	131
	371	Pineapple.tw.	1295
	372	Pinebark extract*.tw.	0
	373	Piper methysticum.tw.	248
	374	Piper nigrum.tw.	438
	375	Piperine.tw.	991
	376	Piscidia piscipula.tw.	8
	377	Polyporus umbellatus.tw.	87
	378	Pomegranate*.tw.	1938
	379	Pr#biotic*.tw.	26259
	380	Prickly pear*.tw.	233
	381	Propolis.tw.	2963
	382	Prun*.tw.	9404
	383	Punicaceae.tw.	53
	384	Pycnogenol.tw.	380
	385	Quercetin.tw.	16178
	386	Radish*.tw.	2837
	387	Rainforest lime*.tw.	0
	388	Raisin tree*.tw.	7
	389	Rapeseed.tw.	3534
	390	Raphanus.tw.	1222
	391	Raspberr*.tw.	1807
	392	Reishi.tw.	184
	393	Resveratrol.tw.	11749
	394	Rhodiola.tw.	814
	395	Ribes.tw.	499
	396	Rooibos.tw.	224
	397	Rosa.tw.	2684
	398	Rose*.tw.	93851
	399	Rosmarinus.tw.	828
	400	Royal jelly.tw.	800

	401	Rubus.tw.	1004
	402	Saccharomyces.tw.	70375
	403	Sage.tw.	3604
	404	Salix.tw.	1412
	405	Salvia officinalis.tw.	541
	406	Sambucus nigra.tw.	715
	407	Schi#andra.tw.	1029
	408	Scutellaria lateriflora.tw.	44
	409	Serrapeptase.tw.	19
	410	Serratiopeptidase.tw.	73
	411	Shiitake mushroom*.tw.	266
	412	Silybum marianum.tw.	721
	413	Siraitia grosvenorii.tw.	85
	414	Skullcap.tw.	166
	415	Stinging nettle*.tw.	182
	416	Sugar beet*.tw.	2699
	417	Syzygium aromaticum.tw.	323
	418	Taraxacum.tw.	615
	419	Tea.tw.	28752
	420	Terminalia ferdinandiana.tw.	18
	421	Thioctic acid.tw.	546
	422	Tilia.tw.	359
	423	Trametes versicolor.tw.	854
	424	Turkey tail.tw.	11
	425	Turmeric.tw.	2745
	426	Turnera diffusa.tw.	46
	427	Ubidecarenone.tw.	65
	428	Ubiquinone.tw.	6830
	429	Urtica dioica.tw.	510
	430	Vaccinium macrocarpon.tw.	242
	431	Valerian.tw.	518
	432	Vanilla.tw.	713
	433	Verbascum thapsis.tw.	50
	434	Verbena.tw.	228
	435	Vervain.tw.	16
	436	Vitis.tw.	4367
	437	Watermelon.tw.	1743
	438	Wheatgrass.tw.	279
	439	Willow bark.tw.	142
	440	Withania somnifera.tw.	905
	441	Wolf berr*.tw.	4
	442	Yam.tw.	945
	443	Yerba mate.tw.	236
	444	Zhu ling.tw.	8
	445	Zingiber officinale.tw.	1139
	446	Zizyphus.tw.	257
	447	or/28-447	1290272
Minerals	448	exp Bicarbonates/	24402
	449	exp Calcium compounds/	73245
	450	exp Chlorides/	132839
	451	exp Citrates/	22772

	452	exp Magnesium compounds/	16515
	453	exp Potassium compounds/	29424
	454	exp Sodium compounds/	100477
	455	exp Zinc compounds/	12282
	456	Calcium/	263307
	457	Chromium compounds/	1221
	458	Chromium/	14019
	459	Copper/	66666
	460	Electrolytes/	24900
	461	Iodides/	9826
	462	Iodine compounds/	887
	463	Iodine/	24844
	464	Iron, dietary/	2843
	465	Iron/	91615
	466	Magnesium/	66406
	467	Manganese compounds/	3620
	468	Manganese/	23947
	469	Phosphates/	61868
	470	Potassium/	100207
	471	Selenium compounds/	3705
	472	Selenium/	20110
	473	Sodium/	104579
	474	Zinc/	58231
	475	Bicarbonate*.tw.	28596
	476	Calcium.tw.	358331
	477	Chloride*.tw.	140374
	478	Chromium.tw.	23102
	479	Citrate*.tw.	43511
	480	Copper.tw.	97577
	481	Dipotassium.tw.	621
	482	Electrolyte*.tw.	69795
	483	Ferric.tw.	18424
	484	Ferrous.tw.	12178
	485	Iodide.tw.	32008
	486	Iodine.tw.	46868
	487	Iron.tw.	175753
	488	Mag Phos.tw.	0
	489	Magnesium.tw.	55454
	490	Manganese.tw.	31437
	491	Mineral.tw.	104134
	492	Minerals.tw.	20772
	493	Monopotassium.tw.	141
	494	Phosphate*.tw.	250664
	495	Potassium.tw.	131743
	496	Salt.tw.	135409
	497	Selenate.tw.	1553
	498	Selenium.tw.	26851
	499	Selenomethionine.tw.	2439
	500	Sodium.tw.	336448
	501	Zinc.tw.	110543
	502	or/448-501	2138558

Macro-nutrients Part 1	503	exp beta-Glucans/	9183
	504	exp Carbohydrates/	1520975
	505	exp Fruit/	98682
	506	exp Sweetening agents/	222359
	507	Dietary fats/	47248
	508	Honey/	3701
	509	Inositol/	7495
	510	Lecithins/	1686
	511	Micronutrients/	5440
	512	Nutrients/	954
	513	Plants, edible/	5925
	514	(Diet* adj3 (carbohydrate* or fat* or fibre or fiber or sugar*)).tw.	71890
	515	(Edible adj3 (herb* or plant*)).tw.	1764
	516	(Intake adj3 (carbohydrate* or fat* or fibre or fiber or fluid or food or water)).tw.	75503
	517	Beta-glucan*.tw.	5705
	518	Fruit*.tw.	100060
	519	Glucose.tw.	444489
	520	Honey.tw.	10275
	521	Inositol.tw.	35875
	522	Inulin.tw.	7989
	523	Lecithin.tw.	9124
	524	M#cronutrient*.tw.	21441
	525	Nutrient*.tw.	133944
	526	Nutrition.tw.	146434
	527	Phosphatidylcholine.tw.	30063
	528	Sweetener*.tw.	3744
	529	Trace element*.tw.	17852
	530	Vegetable*.tw.	51950
	531	or/503-530	2286651
Macro-nutrients Part 2	532	exp Milk proteins/	36432
	533	5-Hydroxytryptophan/	4078
	534	Amino acids/	134965
	535	Arginine/	41891
	536	Citrulline/	4071
	537	Cysteine/	36485
	538	Dietary proteins/	36879
	539	gamma-Aminobutyric acid/	37519
	540	Glutamine/	17007
	541	Glutathione/	54894
	542	Grain proteins/	13
	543	Griffonia/	25
	544	Leucine/	26670
	545	Methionine/	25748
	546	Nuts/	3073
	547	Plant proteins, dietary/	1774
	548	Selenomethionine/	1590
	549	Taurine/	9605
	550	(Protein* adj2 (dietary or grain or intake or isolate or milk or nut or plant or rice or whey or vegetable)).tw.	39489
	551	4 Aminobutyric acid.tw.	198

	552	5-Hydroxytryptophan.tw.	3219
	553	Amino acid*.tw.	462393
	554	Arginine.tw.	92974
	555	Citrulline.tw.	5955
	556	Cysteine.tw.	89359
	557	GABA.tw.	51105
	558	Gamma aminobut?ric acid.tw.	23373
	559	Glutamine.tw.	35422
	560	Glutathione.tw.	120917
	561	Griffonia simplicifolia.tw.	720
	562	Leucine.tw.	50860
	563	Methionine.tw.	48255
	564	Nut.tw.	5042
	565	Nuts.tw.	5234
	566	Selenomethionine.tw.	2439
	567	Setria.tw.	2
	568	Sustamine.tw.	3
	569	Taurine.tw.	12759
	570	Theanine.tw.	478
	571	Tryptophan.tw.	46078
	572	or/532-571	1087737
Vitamins	573	exp Cholecalciferol/	26020
	574	exp Vitamin B 6/	15921
	575	exp Vitamin E/	32136
	576	Antioxidants/	113911
	577	Ascorbic acid/	41362
	578	beta Carotene/	7471
	579	Biotin/	13712
	580	Carotenoids/	18343
	581	Folic acid/	26467
	582	Free radical scavengers/	20331
	583	Niacin/	10679
	584	Niacinamide/	12212
	585	Nicotinic acids/	12645
	586	Pantothenic acid/	2899
	587	Phenol/	5417
	588	Polyphenols/	10618
	589	Riboflavin/	8270
	590	Thiamine/	9879
	591	Vitamin A/	23631
	592	Vitamin B 12/	20525
	593	Vitamin B complex/	8482
	594	Vitamin D/	33009
	595	Vitamins/	30311
	596	(Vitamin* adj1 (A or B or B1 or B2 or B3 or B5 or B6 or B7 or B9 or B12 or C or D or D3 or E)).tw.	161577
	597	Antioxidant*.tw.	184791
	598	Ascorbic acid.tw.	30377
	599	Asorbate.tw.	15772
	600	Beta carotene*.tw.	13385
	601	Biotin.tw.	26660

	602	Carotenoid*.tw.	19285
	603	Cholecalciferol.tw.	2347
	604	Cobalamin.tw.	4075
	605	Cyanocobalamin.tw.	1289
	606	Folate.tw.	25125
	607	Folic acid.tw.	19405
	608	Free radical scavenger*.tw.	5590
	609	Methylcobalamin.tw.	675
	610	Niacin.tw.	4705
	611	Niacinamide.tw.	467
	612	Nicotinamide.tw.	19854
	613	Nicotinic acid.tw.	5829
	614	Pantothenate.tw.	1374
	615	Pantothenic acid.tw.	1765
	616	Phenol*.tw.	88630
	617	Polyphenol*.tw.	29030
	618	Pyridoxal.tw.	8222
	619	Pyridoxamine.tw.	1084
	620	Pyrixodine.tw.	5469
	621	Retinal.tw.	146324
	622	Retinol.tw.	13471
	623	Riboflavin.tw.	9688
	624	Thiamin*.tw.	12294
	625	Tocopher#l.tw.	20023
	626	Tocotrienol.tw.	1092
	627	or/573-626	841767
Concepts	628	27 or 447 or 502 or 531 or 572 or 627	6268576
Travel	629	Aerospace medicine/	14727
	630	Air travel/	323
	631	Aircraft/	8599
	632	Aviation/	6042
	633	Pilots/	362
	634	Travel medicine/	657
	635	(Air adj2 (passenger* or transport* or travel*)).tw.	3384
	636	((Air or cabin or civil or commercial) adj5 (pilot* or attendant* or steward* or hostess or crew*)).tw.	1463
	637	((Flight*1 or flying) not (spectro* or TOF or time-of-flight)).tw.	32762
	638	Aeroplane*.tw.	182
	639	Aerospace.tw.	2277
	640	Aircraft*.tw.	6960
	641	Airline*.tw.	2002
	642	Airplane*.tw.	1388
	643	Aviation.tw.	4797
	644	Aviator*.tw.	1003
	645	Emporiatrics.tw.	22
	646	Travel* health.tw.	551
	647	Travel* medicine.tw.	814
	648	or/629-647	60376
Modified SIGN filter	649	exp Clinical trial/	837383
	650	Cross-over studies/	46073
	651	Control group/	1631

	652	Double blind method/	153515
	653	Placebos/	34473
	654	Random allocation/	100553
	655	Single blind method/	27371
		Clinical study.pt.	3341
		Clinical trial.pt.	518157
	658	Clinical trial, phase i.pt.	19369
	659	Clinical trial, phase ii.pt.	31279
	660	Clinical trial, phase iii.pt.	15569
	661	Clinical trial, phase iv.pt.	1757
	662	Controlled clinical trial.pt.	93274
	663	Randomized controlled trial.pt.	490335
	664	Multicenter study.pt.	257481
	665	((Control* or Clinical) adj2 (trial* or study)).tw.	945574
	666	((Cross?over or counter?balance*) adj2 (design or stud* or trial*)).tw.	55143
	667	((Non?random* or random*) adj4 (allocat* or assign* or group*)).tw.	236059
	668	((Singl* or doubl* or treb* or tripl*) adj (blind*3 or mask*3)).tw.	166306
	669	Control group*.tw.	432933
	670	Non?RCT*.tw.	666
	671	Placebo*.tw.	207546
	672	RCT*.tw.	43037
	673	or/649-672	2158044
Final	674	628 and 648 and 673	489

[1] Number of articles identified from inception to 1st October 2019. The search was rerun prior to publication to include up until the 25th of February 2021 however the values in this column have not been updated.
